# Analogs of the carotane antibiotic fulvoferruginin from submerged cultures of a Thai *Marasmius* sp.

**DOI:** 10.3762/bjoc.17.97

**Published:** 2021-06-04

**Authors:** Birthe Sandargo, Leon Kaysan, Rémy B Teponno, Christian Richter, Benjarong Thongbai, Frank Surup, Marc Stadler

**Affiliations:** 1 Microbial Drugs Department, Helmholtz Centre for Infection Research, Inhoffenstr. 7, 38124 Braunschweig, Germany; 2 Technical University of Ilmenau, 98693 Ilmenau, Germany; 3 Department of Chemistry, Faculty of Science, University of Dschang, P.O. Box 67, Dschang, Cameroon,; 4 Institute of Microbiology, Technical University of Braunschweig, Germany

**Keywords:** antibiotics, Basidiomycota, carotane, fulvoferruginin, *Marasmius*, sesquiterpenoid

## Abstract

A recent find of a *Marasmius* species in Northern Thailand led to the isolation of five unprecedented derivatives of the carotane antibiotic fulvoferruginin (**1**), fulvoferruginins B–F (**2**–**6**). The structures of these sesquiterpenoids were elucidated using HRESIMS, 1D and 2D NMR, as well as CD spectroscopy. Assessing the bioactivity, fulvoferruginin emerged as a potent cytotoxic agent of potential pharmaceutical interest.

## Introduction

The family Marasmiaceae (Agaricomycetes, Basidiomycota) presently contains ten genera with *Marasmius* Fr. being the predominant one (currently comprising over 600 recognized species). Most of these fungi are considered saprotrophs, even though some (e.g., *Moniliophthora* species) are plant pathogens [1]. They are rather ubiquitous, but have been frequently overlooked owing to the small dimensions of their basidiomes. As of now, their taxonomy is not well settled, and a world monograph using modern mycological methodology is still not available.

**Figure 1:**
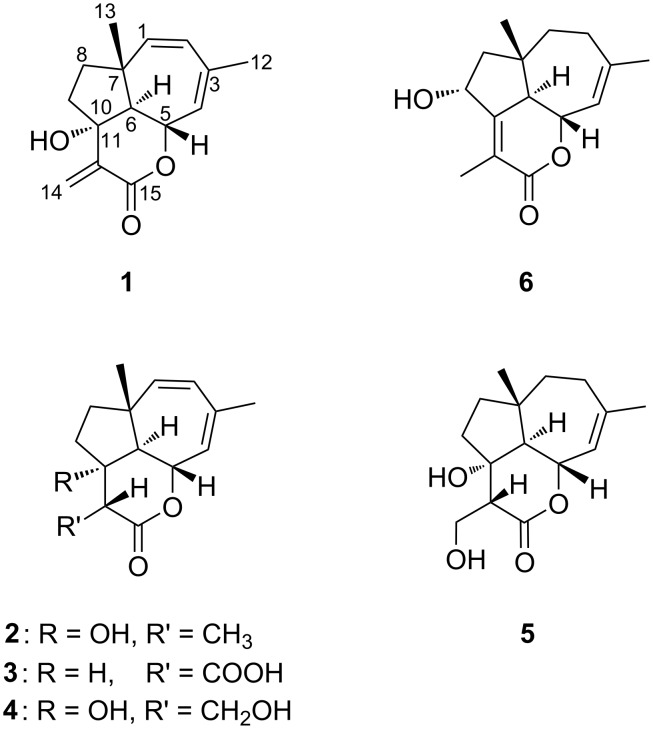
Structures of fulvoferruginin (**1**) and the newly isolated derivatives fulvoferruginins B–F (**2**–**6**).

As of 2006, the family forms a sister taxon to the Omphalotaceae within the suborder of Marasmiineae (Agaricales) [2–3]. Among Basidiomycota, the Marasmiineae are most famous for their abundant chemical diversity. Metabolites with various bioactivities described from *Marasmius* spp. include the cryptoporic acids [4], marasmals, marasmones, and oreadones [5–6], the caryophyllane hebelophyllene C [7], and the carotane fulvoferruginin (**1**) [8] (Figure 1). The latter is the only known secondary metabolite from *M. fulvoferrugineus* Gilliam and displays a modest antibiotic and moderate antifungal activity. A recent find of a *Marasmius* species in Northern Thailand led, however, to the isolation and identification of five novel derivatives of this carotane-type sesquiterpenoid. The present study is dedicated to describing their isolation, and biological and physicochemical characterization.

## Results

### Structure elucidation of the secondary metabolites **2–6**

Preparative HPLC of the supernatant crude extract of a submerged cultivation of *Marasmius* sp. strain MFLUCC 14-0681 in ZM½ media led to the isolation of six carotane sesquiterpenoids, fulvoferruginins A–F (**1**–**6**, Figure 1).

**Figure 2:**
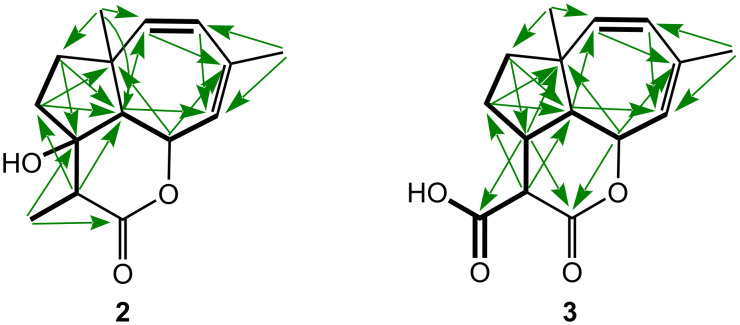
KEY HMBC (arrows) and COSY (thick bonds) correlations of compounds **2** and **3**.

The first isolated metabolite (**2**) showed a protonated molecular ion peak at *m/z* 249.1477 [M + H]^+^ in the HRESIMS, corresponding to the molecular formula C_15_H_20_O_3_ (calcd for C_15_H_21_O_3_^+^, 249.1490). The ^1^H NMR spectrum showed signals for three methyl groups at δ_H_ 0.99 (s, H-13), 1.20 (d, *J* = 7.0 Hz, H-14), and 1.91 (t, *J* = 1.7 Hz, H-12); two methylenes at δ_H_ 1.69 (m, H-8a), 1.85 (m, H-9a), 2.00 (m, H-9b), and 2.05 (m, H-8b) as well as five methines of which one is oxygenated at δ_H_ 4.95 (br d, *J* = 12.1 Hz, H-5) and three are olefinic: δ_H_ 5.59 (d, *J* = 11.2 Hz, H-2), 5.71 (d, *J* = 0.9 Hz, H-4), and 6.09 (d, *J* = 11.2 Hz, H-1). The ^13^C NMR spectrum showed 15 signals including those of a lactone carbonyl at δ_C_ 177.6 (C-15); four olefinic carbons at δ_C_ 126.0 (C-4), 126.5 (C-2), 133.4 (C-3), and 144.0 (C-1); three methyl groups at δ_C_ 8.7 (C-14), 20.2 (C-13), and 28.0 (C-12) (Table 1). The remaining resonances were due to two methylenes at δ_C_ 38.8 (C-8) and 39.2 (C-9); three methines at δ_C_ 46.4 (C-11), 58.4 (C-6), and 77.6 (C-7) as well as two quaternary carbons at δ_C_ 45.6 (C-8) and 82.8 (C-10). Correlating 1D data with 2D NMR experiments (^1^H,^1^H COSY, ^1^H,^13^C HMBC, ^1^H,^1^H ROESY, see Figure 2) led to an underlying structure resembling that of the carotane sesquiterpenoid fulvoferruginin (**1**, [8]) with the only difference being a tertiary methyl group at C-14 (δ_C/H_ 8.7/1.20), instead of an olefin. Compound **2** was therefore named fulvoferruginin B. Subsequently, a minor peak following **2** in the HPLC chromatogram was isolated and identified as fulvoferruginin (**1**), utilizing HRESIMS and 1D/2D NMR experiments (see Table S3 in Supporting Information File 1).

**Table 1:** NMR chemical shifts of compounds **2**–**6** (^1^H NMR (700 MHz) and ^13^C NMR (176 MHz) in methanol-*d*_4_.

Pos.	**2**		**3**		**4**		**5**		**6**	
	^13^C, mult.	^1^H, mult. (*J*, Hz)	^13^C, mult.	^1^H, mult. (*J*, Hz)	^13^C, mult.	^1^H, mult. (*J*, Hz)	^13^C, mult.	^1^H, mult. (*J*, Hz)	^13^C, mult.	^1^H, mult. (*J*, Hz)

1	144.0, CH	6.09, d (11.2)	144.1, CH	6.09, d (11.2)	143.9, CH	6.09, d (11.2)	41.0, CH_2_	1.76, ddd (13.5, 8.1, 3.4)	39.9, CH_2_	1.58, ddd (17.3, 13.6, 3.7)
								1.54, td (13.5, 4.1)		1.78, m
2	126.5, CH	5.59, d (11.2)	126.7, CH	5.60, d (11.2)	126.6, CH	5.60, dd (11.2, 0.6)	31.7, CH_2_	2.12, m	31.1, CH_2_	2.08, dt (17.3, 3.7)
								2.38, m		2.38, m
3	133.4, C_q_		133.9, C_q_		133.5, C_q_		138.1, C_q_		139.0, C_q_	
4	126.0, CH	5.71, d (0.9)	125.6, CH	5.70, dd (2.2, 1.2)	125.7, CH	5.71, dd (2.2, 1.2)	127.0, CH	5.66, dt (2.8, 1.7)	127.1, CH	5.58, br s
5	77.6, CH	4.95, br d (12.1)	79.5, CH	5.04, dt (12.2, 1.9)	77.8, CH	4.96, br d (12.2)	77.6, CH	4.79, br dd (11.4, 1.7)	79.5, CH	4.71, br dd (12.4, 1.8)
6	58.4, CH	2.33, d (12.1)	46.8, CH	2.51, t (12.2)	58.9, CH	2.28, d (12.2)	64.7, CH	1.83, m	53.5, CH	2.56, br dt (12.4, 2.8)
7	45.6, C_q_		45.4, C_q_		45.3, C_q_		44.4, C_q_		40.9, C_q_	
8	38.8, CH_2_	2.05, m	41.1, CH_2_	1.78, m	38.9, CH_2_	1.70, dd (11.8, 6.1)	42.2, CH_2_	1.58, m	51.9, CH_2_	1.52, dd (12.4, 8.9)
		1.69, m				2.06, m		1.84, m		2.18, dd (12.4, 7.6)
9	39.2, CH_2_	1.85, m	31.3, CH_2_	1.61, m	39.6, CH_2_	1.94, dd (13.1, 6.1)	38.6, CH_2_	1.83, m	70.8, CH	4.88, m
		2.00, m		2.08, m		2.17, m		2.18, m		
10	82.8, C_q_		39.3, CH	2.69, m	82.5, C_q_		83.7, C_q_		161.7, C_q_	
11	46.4, CH	2.72, q (7.0)	52.7, CH^a^		53.4, CH	2.71, dd (6.4, 4.0)	54.2, CH	2.66, dd (6.4, 4.0)	123.9, C_q_	
12	28.0, CH_3_	1.91, t (1.7)	28.1, CH_3_	1.91, t (1.7)	28.0, CH_3_	1.91, t (1.7)	29.0, CH_3_	1.80, dt (2.0, 1.2)	28.9, CH_3_	1.80, s
13	20.2, CH_3_	0.99, s	21.2, CH_3_	1.03, s	20.1, CH_3_	1.00, s	17.8, CH_3_	1.00, s	18.2, CH_3_	0.85, s
14	8.7, CH_3_	1.20, d (7.0)	171.9, C_q_		59.0, CH_2_	3.94, dd (11.4, 4.0)	59.2, CH_2_	3.93, dd (11.4, 4.0)	12.1, CH_3_	1.93, dd (2.8, 1.0)
						4.10, dd (11.4, 6.4)		4.10, dd (11.4, 6.4)		
15	177.6, C_q_		173.0, C_q_		175.6, C_q_		175.7, C_q_		169.6, C_q_	

^a 13^C NMR chemical shift extracted from HMBC spectrum. CH verified by measuring **3** in chloroform-*d* (see Table S1 in Supporting Information File 1).

**Figure 3:**
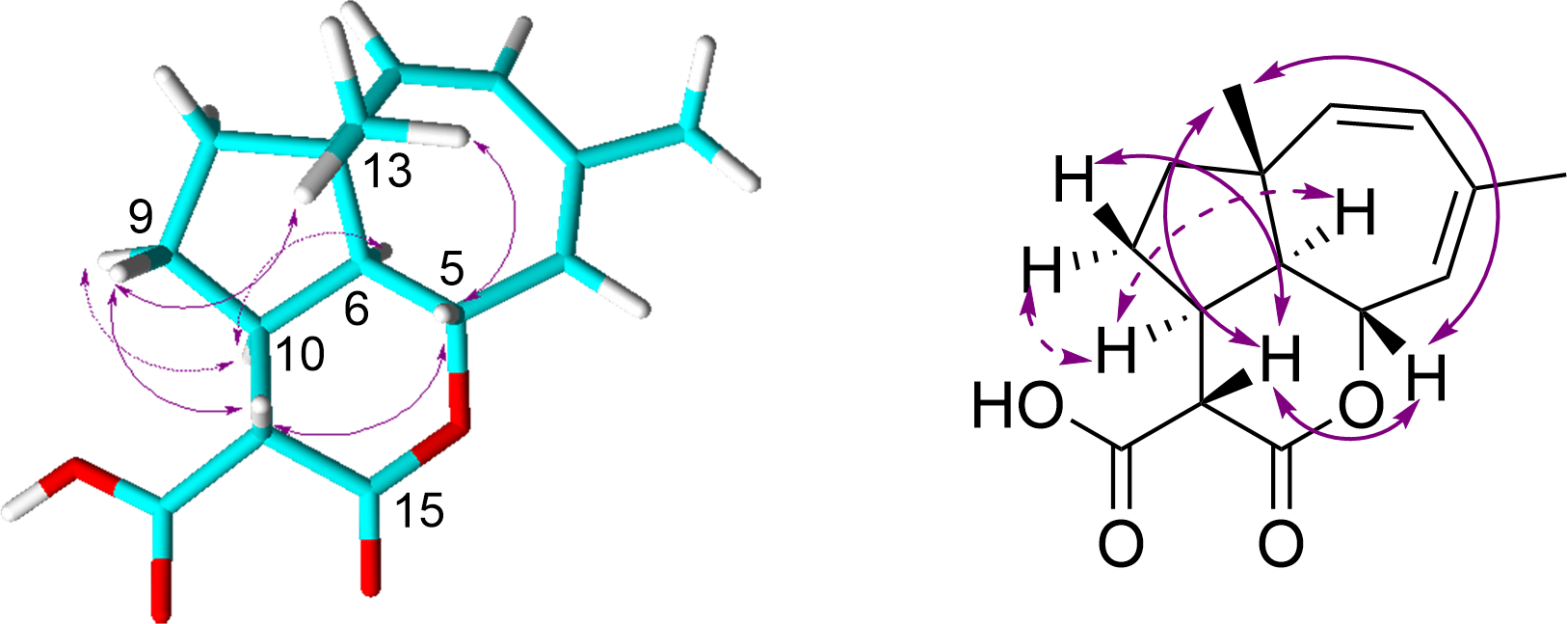
Key ROESY correlations of metabolite **3**.

The methyl group C-14 of fulvoferruginin B (**2**) results in an additional stereocenter at C-11. The proton at C-11 (δ_H_ 2.72) exhibited ^1^H,^1^H ROESY correlations with H-5 (δ_H_ 4.95) and H_3_-13 (δ_H_ 0.99) indicating that they are on the same face of the molecule. These ROESY correlations are otherwise identical to those observed for fulvoferruginin (**1**). The relative stereochemistry at C-6 and C-10 was further confirmed through comparison with ROESY correlations of metabolite **3** (Figure 3).

A third isolated compound (**3**) with a molecular formula of C_15_H_18_O_4_, derived from a protonated molecular ion peak of *m/z* 263.1276 [M + H]^+^, was named fulvoferruginin C (**3**). It differed from compound **2** only in the presence of a carboxyl group (δ_C_ 171.9) instead of the tertiary methyl C-14, and a methine (C-10, δ_C_ 39.3) instead of a hydroxylated quaternary carbon. The presence of the methine C-11 was confirmed by measuring 1D/2D NMR spectra of **3** in chloroform-*d* (see Table S1 in Supporting Information File 1). The corresponding proton H-11 (δ_H_ 3.38) forms again a spin system with H-5 (δ_H_ 4.89) and H-13 (δ_H_ 1.01) in the COSY spectrum (Figure 2). Furthermore, the relative configurations of C-6 and C-10 were elucidated by the ROESY correlation (Figure 3) depicted between H-6 (δ_H_ 2.57) and H-10 (δ_H_ 2.75). All these correlations also support the structure of compound **2**.

Metabolite **4**, trivially named fulvoferruginin D, is structurally similar to compound **2**. Its molecular formula C_15_H_20_O_4_ was deduced from the HRESIMS which exhibited ions at *m/z* 265.1434 [M + H]^+^ (calcd for C_15_H_21_O_4_^+^, 265.1439) and 287.1252 [M + Na]^+^ (calcd for C_15_H_20_NaO_4_^+^, 287.1254). The examination of the NMR spectroscopic data revealed that C-14 is hydroxylated in compound **4** (δ_C_ 59.0) as compared to fulvoferruginin B (**2**). This was further confirmed by HMBC correlations observed between the hydroxymethylene protons [δ_H_ 3.94 (dd, *J* = 11.4, 4.0 Hz, H-14a) and 4.10 (dd, *J* = 11.4, 6.4 Hz, H-14b)] and carbons at δ_C_ 53.4 (C-11), 82.5 (C-10), and 175.6 (C-15) (see Table 1 and Supporting Information File 1).

Fulvoferruginin E (**5**), isolated as a clear solid, shares the same molecular formula C_15_H_22_O_4_ as compound **2**, as deduced from the HRESIMS spectrum also exhibiting a protonated molecular ion peak at *m/z* 267.1589 [M + H]^+^. Its 1D/2D NMR spectroscopic data are similar to those of metabolite **4**, with the only striking variation being the replacement of two olefinic methines (C-1/C-2) with methylenes (C-1: δ_C_ 41.0, C-2: δ_C_ 31.7). The absence of this double bond has previously been observed in the structurally related hercynolactone from the liverworts *Barbilophozia lycopodioides* and *B. hatcheri* [9].

To add to this, another metabolite, fulvoferruginin F (**6**), with a molecular formula of C_15_H_20_O_3_ was isolated that is also lacking the double bond at C-1/C-2. It further possesses an additional double bond between C-10/C-11, just like hercynolactone. However, NMR data analysis disclosed that the position C-9 (δ_C_ 70.8) is hydroxylated. The ROESY spectrum indicates H-9 (δ_H_ 4.88) to be cofacial with H-5 and H-13. Thus, fulvoferruginin F could also be named 9-hydroxyhercynolactone [9].

Assessing the bioactivity of the fulvoferruginins A–F (**1–6**), aside from the known antifungal activity of fulvoferruginin (**1**), no other antimicrobial activities were observed (Table S2 in Supporting Information File 1). All metabolites were also tested against the murine fibroblast cell line L929 and the cervix carcinoma cell line KB3.1. While all metabolites exhibited very weak cytotoxic effects at the highest concentration, only **2**, **4**, and **6** displayed mild cytotoxicity, allowing for a determination of IC_50_ values, which ranged from 9.5–32 µg/mL. To our surprise, fulvoferruginin (**1**) displayed greater cytotoxic effects than previously reported (though for other cell lines) [8], which led us to assess its cytotoxicity further against different carcinoma cell lines. The IC_50_ values of compound **1** range from 0.06–0.7 µg/mL for all tested cell lines (Table S2 in Supporting Information File 1).

## Discussion

The lack of bioactivity for metabolites **2**–**6** can be attributed to an absence of the α-methylene lactone unit present in fulvoferruginin (**1**). Nevertheless, the cytotoxicity detected here for fulvoferruginin shows that re-evaluating the bioactivity of previously isolated basidiomycete metabolites in different bioassays can lead to unexpected results of potential pharmaceutical interest.

Klein et al. obtained a crystal structure of fulvoferruginin (**1**) [8], as did Huneck et al. for hercynolactone [9], verifying their relative configuration. Huo et al. have further confirmed the absolute configuration of compound **1** by utilizing the CD exciton chirality method. Additionally, our recorded CD spectra (Figure S1 in Supporting Information File 1) of metabolites **3** and **4**, are in close agreement with **1**. As the metabolites **2–6** displayed analogous relative stereochemistry and optical rotations, we presume that the new compounds arise from the same biosynthetic genes as the parent compound, and postulate that the congeners should also have the same absolute configuration as compound **1** [10].

For a long time, fulvoferruginin was only known to be produced by a strain of *M. fulvoferrugineus* found in Northern America, but there is a recent report on the occurrence of fulvoferruginin (**1**) in a basidiomycete collected in China. This strain was tentatively identified as *Gymnopus* sp. through analysis of its internal transcribed spacer (ITS1-5.8s) rDNA region [10]. Today, *Gymnopus* belongs to the Omphalotaceae and some *Marasmius* species have been reassigned to *Gymnopus*, as have certain *Gymnopus* spp. to genera like *Marasmiellus*. However, intensive phylogenetic studies on these genera remain to be conducted. A macroscopic differentiation to *Marasmius* is difficult and a taxonomic classification based on ITS alone has proven to be insufficient for these genera [11]. In addition, the recent finding that multiple copies of the rDNA can be present in one and the same genome of certain fungi, leading to up to more than 10% deviations [12], makes us suspicious about the validity of the previous classification of the Chinese “*Gymnopus*” species.

Taking also other marker loci into consideration, as is required for publishing a new fungal species [12], *Marasmius* sp. MFLUCC14-0681 currently displays the largest identity (BLASTn) to a European *M. haematocephalus* (98.9%), a Brazilian *M. conchiformis*, and *M. purpureostriatus*. Nevertheless, all species are genetically distinct. Considering other rDNA regions currently leads to more questions than answers regarding the phylogeny and clearly shows the need for further taxonomic re-evaluation.

## Experimental

### General experimental procedures

An Agilent 1200 series HPLC-UV system (Santa Clara, CA, USA) with an ESI-TOF-MS (MaXis, Bruker, Bremen, Germany) [column 2.1 × 50 mm, 1.7 µm, C18 Acquity UPLC BEH (Waters, Eschborn, Germany)], with deionized water + 0.1% formic acid (solvent A) as well as acetonitrile + 0.1% formic acid (solvent B) and a gradient of 5% B for 0.5 min increasing to 100% B in 19.5 min, maintaining 100% B for 5 min, flow rate: 0.6 mL/min, UV detection 200–600 nm, was used to obtain the HRESIMS data. NMR spectra were acquired on a Bruker Avance III 700 MHz spectrometer (Bremen, Germany) equipped with a 5 mm TCI cryoprobe (^1^H NMR (700 MHz), ^13^C NMR (175 MHz)). Optical rotations of the metabolites were measured with an MCP 150 polarimeter (Anton Paar, Graz, Austria). The CD spectra were obtained from a JASCO spectropolarimeter, model J-815 (Pfungstadt, Germany). The UV spectra were recorded on a Shimadzu UV–vis spectrophotometer UV-2450 (Duisburg, Germany).

### Isolation and identification of fungal material

Basidiomes of strain MFLUCC 14-0681 were collected and isolated by B.T. close to the Mushroom Research Center in Chiang Mai Province, Thailand (http://www.mushroomresearchcentre.com) on August 11, 2014. The species was identified as a *Marasmius* sp. by mycologists B.T. and M.S. based on its morphological characteristics and cultures were verified by rDNA sequence comparison [5.8S gene region, the internal transcribed spacer 1 and 2 (ITS) and the large subunit (LSU)]. Sequence data are deposited with GenBank, under accession number MN150126 for ITS and MN150183 for LSU. The collected, dried specimen as well as corresponding cultures were deposited at the mycological herbarium of Mae Fah Luang University, Chiang Rai, Thailand, accession number MFLUCC 14-0681.

The DNA extraction was performed using an EZ-10 Spin Column Genomic DNA Miniprep kit (Bio Basic Canada Inc., Markham, Ontario, Canada) following the manufacturer’s protocol. A Precellys 24 homogenizer (Bertin Technologies, France) at 6000 rpm for 2 × 40 s was used for cell disruption. DNA regions were amplified using standard primers following our previously published protocols [13]. For both DNA regions, the PCR products were purified utilizing the Nucleo Spin^®^ Gel and PCR Clean-up kit (Macherey-Nagel, Düren, Germany). Sequencing of the PCR products was carried out at the Department of Genome Analytics of the Helmholtz Centre for Infection Research, Braunschweig, Germany.

### Fermentation and isolation of metabolites **1–6**

Cultures of *Marasmius* sp. strain MFLUCC 14-0681 were maintained on YMG agar, as described by Klein et al. [8]. A 100 mL seed culture of the strain in ZM½ medium [14] was used to inoculate a 5 L batch fermentation in 25 500 mL Erlenmeyer flasks with ZM½ media for 15 days at 140 rpm and 23 °C. Afterwards, the fermentation broth was filtered and the supernatant extracted with 2% Amberlite^TM^ XAD 16N (Rohm & Haas, Frankfurt a. M., Germany), subsequently resuspended in acetone and evaporated to dryness, resulting in 2.5 g of crude extract. This supernatant crude extract was filtered using a SPME Strata^TM^-X 33 u Polymeric RP cartridge (Phenomenex, Inc., Aschaffenburg, Germany) and subsequently pre-fractionated utilizing RP-HPLC with a Gilson PLC 2250 purification system (Middleton, WI, USA) and a VP Nucleodur 100-5 C_18_ ec 250 × 40 mm, 7 µm column (Macherey-Nagel, Düren, Germany). Acetonitrile + 0.1% formic acid and deionized water + 0.1% FA served as mobile phase, running a gradient of 10 min at 5% acetonitrile + 0.1% FA, then increasing to 100% within 70 min, flow rate: 40 mL/min, UV detection at 200–600 nm. Pre-fractions were further separated using a Luna C18(2) 250 × 21 mm, 7 μm column (Phenomenex, Aschaffenburg, Germany) and deionized water + 0.05% TFA (solvent A) and acetonitrile + 0.05% TFA (solvent B) as mobile phase and a flow rate of 18 mL/min. Gradients were established individually for each pre-fraction as follows: a pre-fraction at 36 to 37 minutes was further separated with a gradient of 20–50% solvent B within 60 minutes, resulting in a peak of metabolite **4** (32 mg) at 27–27.5 min. The pre-fraction at 38 minutes was separated with a gradient from 20–70% B within 60 minutes of which a peak at 24–25 min resulted in metabolite **5** (1.6 mg). A pre-fraction from 42–43 min was further separated with a gradient from 30–60% B within 60 min, resulting in a major peak at 26–27 min being fulvoferruginin B (**2**, 16 mg) as well as a minor peak at 27–28 min, being fulvoferruginin (**1**, 9.8 mg). Yet another pre-fraction at 45 min was further separated using a gradient of 30% solvent B for 10 minutes then increasing to 75% B within 45 min. A peak at 30–30.5 min resulted in fulvoferruginin C (**3**, 28 mg). The pre-fraction peak at 41–42 minutes was further separated with a gradient of 30% solvent B for 20 min, then increased from 30 to 50% B within 40 min using an XBridge^TM^ Trifunctional C18, 250 × 19 mm, 135 Å, 5 μm column (Waters, Eschborn, Germany) as stationary phase, resulting in a peak of metabolite **6** (1.2 mg) at 21–21.5 minutes.

Fulvoferruginin (**1**): colorless solid; [α]_D_^20^ −66 (*c* 1, MeOH); UV (MeOH) λ_max_ (PDA) 200, 247 nm; ^1^H (700 MHz) and ^13^C NMR (175 MHz) data in CD_3_OD are collected in Table 1 and copies of spectra are provided in Supporting Information File 1.

Fulvoferruginin B (**2**): faint yellow solid; [α]_D_^20^ +14 (*c* 1, MeOH); UV (MeOH) λ_max_ (log ε) 200 (3.7), 249 (3.5) nm; ^1^H NMR and ^13^C NMR data (^1^H 700 MHz, ^13^C 175 MHz) in CD_3_OD: see Table 1; HRMS–ESI (*m*/*z*): [M + H]^+^ calcd for C_15_H_21_O_3_^+^, 249.1477; found, 249.1490.

Fulvoferruginin C (**3**): colorless solid; [α]_D_^20^ +50 (*c* 1, MeOH); UV (MeOH) λ_max_ (log ε) 200 (3.4), 249 (3.7) nm; ^1^H (700 MHz) and ^13^C NMR (175 MHz) data in CD_3_OD are collected in Table 1, data recorded in CDCl_3_ (Table S1) and copies of spectra are collected in Supporting Information File 1; HRMS–ESI (*m*/*z*): [M + H]^+^ calcd for C_15_H_18_O_4_^+^, 263.1283; found, 263.1276.

Fulvoferruginin D (**4**): light yellow solid; [α]_D_^20^ +19 (*c* 1, MeOH); UV (MeOH) λ_max_ (log ε) 201 (4.4), 249 (4.2) nm; ^1^H (700 MHz) and ^13^C NMR (175 MHz) data in CD_3_OD are collected in Table 1 and copies of spectra are collected in Supporting Information File 1; HRMS–ESI (*m*/*z*): [M + H]^+^ calcd for C_15_H_21_O_4_^+^, 265.1439; found, 265.1434.

Fulvoferruginin E (**5**): colorless solid; [α]_D_^20^ +30 (*c* 1, MeOH); UV (MeOH) λ_max_ (log ε) 200 (3.9), 223 (3.2) nm; ^1^H (700 MHz) and ^13^C NMR (175 MHz) data in CD_3_OD are collected in Table 1 and copies of spectra are collected in Supporting Information File 1; HRMS–ESI (*m*/*z*): [M + H]^+^ calcd for C_15_H_23_O_4_^+^, 267.1596; found, 267.1589.

Fulvoferruginin F/9-hydroxy-hercynolactone (**6**): colorless solid; [α]_D_^20^ +27 (*c* 1, MeOH); UV (MeOH) λ_max_ (log ε) 201 (4.2), 232 (4.0) nm; ^1^H (700 MHz) and ^13^C NMR (175 MHz) data in CD_3_OD are collected in Table 1 and copies of spectra are collected in Supporting Information File 1; HRMS–ESI (*m*/*z*): [M + H]^+^ calcd for C_15_H_21_O_3_^+^, 249.1490; found, 249.1480.

### Antimicrobial activity

The minimum inhibitory concentrations (MIC) of metabolites **1**–**6** were assessed using a serial dilution assay in 96-well microtiter plates with YM6.3 media (10 g/L malt extract, 4 g/L glucose, 4 g/L yeast extract, pH 6.3) for filamentous fungi and yeasts, and with BD Difco^TM^ Mueller Hinton Broth for bacteria. The antimicrobial assays were performed as previously described [13].

### Cytotoxicity assay

The cytotoxicity was assessed in vitro against mouse fibroblast cell line L929, cervix carcinoma cell line KB3.1, ovarian cancer cell line SKOV-3, human adenocarcinoma cell lines MCF-7 and PC-3, and human epidermoid carcinoma cell line A431, using the MTT (3-(4,5-dimethylthiazol-2-yl)-2,5-diphenyltetrazolium bromide) test in 96-well plates [15].

## Supporting Information

File 1 HRESIMS profiles and copies of NMR spectra for compounds **1**–**6** in CD_3_OD, and for metabolite **3** also in CDCl_3_; minimum inhibitory concentrations (MIC) of **1**–**6** for bacteria, yeasts and fungi as well as half inhibitory concentrations (IC_50_) for different cell lines.
