# Supplementary material for: Analogs of the carotane antibiotic fulvoferruginin from submerged cultures of a Thai Marasmius sp
Source: Beilstein J Org Chem. 2021 Jun 4;17:1385–91. doi: 10.3762/bjoc.17.97 (PMC8182674; doi:10.3762/bjoc.17.97)

BEILSTEIN JOURNAL OF ORGANIC CHEMISTRY

Supporting Information

for

Analogs of the carotane antibiotic fulvoferruginin from submerged cultures of a Thai *Marasmius* sp.

Birthe Sandargo, Leon Kaysan, Rémy B. Teponno, Christian Richter, Benjarong Thongbai, Frank Surup and Marc Stadler

Beilstein J. Org. Chem. **2021,** *17,* 1385–1391. doi:10.3762/bjoc.17.97

HRESIMS profiles and copies of NMR spectra for compounds 1–6 in CD_3OD, and for metabolite 3 also in $CDCl_3$; minimum inhibitory concentrations (MIC) of 1–6 for bacteria, yeasts and fungi as well as half inhibitory concentrations (IC_{50}) for different cell lines

License and Terms: This is a supporting information file under the terms of the Creative Commons Attribution License (https://creativecommons.org/licenses/by/4.0). Please note that the reuse, redistribution and reproduction in particular requires that the author(s) and source are credited and that individual graphics may be subject to special legal provisions.
The license is subject to the *Beilstein Journal of Organic Chemistry* terms and conditions: (https://www.beilstein-journals.org/bjoc/terms)

Table S1. NMR chemical shifts (^{1}H 700 MHz, ^{13}C 176 MHz) of Fulvoferruginin C (**3**) in chloroform-*d*. S4

Table S2. Minimum inhibitory concentration (MIC) for bacteria, yeasts and fungi and half-inhibitory concentration (IC_{50}) for cell lines, in µg/ml. S5

Table S3. NMR chemical shifts (^{1}H 500 MHz, ^{13}C 125 MHz) of Fulvoferruginin (A) (**1**) in methanol-d_4. .... S6

Figure S1. ECD spectra of Fulvoferruginin (**1**), Fulvoferruginin C (**3**) and D (**4**) in methanol. S7

HRESIMS of Fulvoferruginin A (**1**).......... S8

- ^{1}H NMR spectrum (500 MHz, methanol-d_4) of Fulvoferruginin (A) (**1**). S9
- ^{13}C NMR spectrum (125 MHz, methanol-d_4) of Fulvoferruginin A (**1**). S10
- HSQC-dept NMR spectrum (500 MHz, methanol-d_4) of Fulvoferruginin A (**1**). S11
- HMBC NMR spectrum (500 MHz, methanol-d_4) of Fulvoferruginin A (**1**). S12
- COSY NMR spectrum (500 MHz, methanol-d_4) of Fulvoferruginin A (**1**). S13
- ROESY NMR spectrum (500 MHz, methanol-d_4) of Fulvoferruginin A (**1**). S14

HRESIMS of Fulvoferruginin B (**2**).......... S15

- ^{1}H NMR spectrum (700 MHz, methanol-d_4) of Fulvoferruginin B (**2**).......... S16
- ^{13}C NMR spectrum (176 MHz, methanol-d_4) of Fulvoferruginin B (**2**). S17
- HSQC-dept NMR spectrum (700 MHz, methanol-d_4) of Fulvoferruginin B (**2**). S18
- HMBC NMR spectrum (700 MHz, methanol-d_4) of Fulvoferruginin B (**2**). S19
- COSY NMR spectrum (700 MHz, methanol-d_4) of Fulvoferruginin B (**2**). S20
- ROESY NMR spectrum (700 MHz, methanol-d_4) of Fulvoferruginin B (**2**). S21

HRESIMS of Fulvoferruginin C (**3**). S22

- ^{1}H NMR spectrum (700 MHz, methanol-d_4) of Fulvoferruginin C (**3**). S23
- ^{13}C NMR spectrum (176 MHz, methanol-d_4) of Fulvoferruginin C (**3**). S24
- HSQC-dept NMR spectrum (700 MHz, methanol-d_4) of Fulvoferruginin C (**3**). S25
- HMBC NMR spectrum (700 MHz, methanol-d_4) of Fulvoferruginin C (**3**). S26
- COSY NMR spectrum (700 MHz, methanol-d_4) of Fulvoferruginin C (**3**). S27
- ROESY NMR spectrum (700 MHz, methanol-d_4) of Fulvoferruginin C (**3**). S28
- ^{1}H NMR spectrum (700 MHz, chloroform-*d*) of Fulvoferruginin C (**3**).......... S29
- ^{13}C NMR spectrum (176 MHz, chloroform-*d*) of Fulvoferruginin C (**3**). S30
- HSQC-dept NMR spectrum (700 MHz, chloroform-*d*) of Fulvoferruginin C (**3**). S31
- HMBC NMR spectrum (700 MHz, chloroform-*d*) of Fulvoferruginin C (**3**). S32
- COSY NMR spectrum (700 MHz, chloroform-*d*) of Fulvoferruginin C (**3**). S33
- ROESY NMR spectrum (700 MHz, chloroform-*d*) of Fulvoferruginin C (**3**). S34

HRESIMS of Fulvoferruginin D (**4**). S35
^{1}H NMR spectrum (700 MHz, methanol-d_4) of Fulvoferruginin D (**4**). S36
^{13}C NMR spectrum (176 MHz, methanol-d_4) of Fulvoferruginin D (**4**). S37
HSQC-dept NMR spectrum (700 MHz, methanol-d_4) of Fulvoferruginin D (**4**). S38
HMBC NMR spectrum (700 MHz, methanol-d_4) of Fulvoferruginin D (**4**). S39
COSY NMR spectrum (700 MHz, methanol-d_4) of Fulvoferruginin D (**4**). S40
ROESY NMR spectrum (700 MHz, methanol-d_4) of Fulvoferruginin D (**4**). S41
HRESIMS of Fulvoferruginin E (**5**). S42
^{1}H NMR spectrum (700 MHz, methanol-d_4) of Fulvoferruginin E (**5**). S43
^{13}C NMR spectrum (176 MHz, methanol-d_4) of Fulvoferruginin E (**5**). S44
HSQC-dept NMR spectrum (700 MHz, methanol-d_4) of Fulvoferruginin E (**5**). S45
HMBC NMR spectrum (700 MHz, methanol-d_4) of Fulvoferruginin E (**5**). S46
COSY NMR spectrum (700 MHz, methanol-d_4) of Fulvoferruginin E (**5**). S47
ROESY NMR spectrum (700 MHz, methanol-d_4) of Fulvoferruginin E (**5**). S48
HRESIMS of Fulvoferruginin F (**6**). S49
^{1}H NMR spectrum (700 MHz, methanol-d_4) of Fulvoferruginin F (**6**). S50
^{13}C NMR spectrum (176 MHz, methanol-d_4) of Fulvoferruginin F (**6**). S51
HSQC-dept NMR spectrum (700 MHz, methanol-d_4) of Fulvoferruginin F (**6**). S52
HMBC NMR spectrum (700 MHz, methanol-d_4) of Fulvoferruginin F (**6**). S53
COSY NMR spectrum (700 MHz, methanol-d_4) of Fulvoferruginin F (**6**). S54
ROESY NMR spectrum (700 MHz, methanol-d_4) of Fulvoferruginin F (**6**). S55

Table S1. NMR chemical shifts (^1H 700 MHz, ^{13}C 176 MHz) of Fulvoferruginin C (**3**) in chloroform-*d*.

Pos.	δ_C	mult	δ_H	mult (*J*, Hz)
1	142.2	CH	6.04	d (11.2)
2	125.9	CH	5.57	d (11.2)
3	132.8	C		
4	123.7	CH	5.72	d (0.6)
5	78.1	CH	4.89	br d (12.1)
6	45.1	CH	2.57	t (12.1)
7	43.9	C		
8	39.8	CH_2	1.77	m
9	30.4	CH_2	1.66	m
			2.19	m
10	37.7	CH	2.75	m
11	51.6	CH	3.38	d (13.3)
12	27.7	CH_3	1.91	m (1.7)
13	20.9	CH_3	1.01	s
14	172.1	C		
15	170.5	C		

Table S2. Minimum inhibitory concentration (MIC) for bacteria, yeasts and fungi and half-inhibitory concentration (IC_{50}) for cell lines, in µg/ml.

A total of 2 µL and 20 µL of a 1 mg/mL stock solution (6.7 and 67 µg/mL) of **1**–**6** were tested. MeOH (20 µL) served as negative control and showed no inhibitory effects. Oxytetracycline served as a reference antimicrobial for bacteria; Nystatin for yeasts and filamentous fungi; Epothilon B was used as positive control for cell lines.

	MIC (µg/ml)						
**Organism**	**1**	**2**	**3**	**4**	**5**	**6**	**Ref.**
Schizos. pombe DSM70572	66.7	-	-	-	-	-	16.7
Mucor hiemalis DSM2656	16.7	-	-	-	-	-	16.7
Candida albicans DSM1665	8.3	-	-	-	-	-	16.7
Rhodoturula glutinis DSM10134	33.3	-	-	-	-	-	16.7
Micrococcus luteus DSM1790	-	-	-	-	-	-	0.5
Staph.aureus DSM346	-	-	-	-	-	-	8.3
Bacillus subtilis DSM10	-	-	-	-	-	-	8.3
Escherichia coli DSM1116	-	-	-	-	-	-	4.2
Pichia anomala DSM6766	33.3	-	-	-	-	-	16.7
	IC_{50} (µg/ml)						
**Cell line**	**1**	**2**	**3**	**4**	**5**	**6**	**Ref.**
L929	0.6	*	*	9.5	*	*	0.00062
KB3.1	0.7	23	*	20	*	32	0.00003
A549	0.5	n.t.	n.t.	n.t.	n.t.	n.t.	0.000016
A431	0.06	n.t.	n.t.	n.t.	n.t.	n.t.	0.000048
SKOV-3	0.7	n.t.	n.t.	n.t.	n.t.	n.t.	0.00013
MCF-7	0.3	n.t.	n.t.	n.t.	n.t.	n.t.	0.000072
PC-3	0.1	n.t.	n.t.	n.t.	n.t.	n.t.	n.t.

* showed cytotoxic effects, but no IC_{50} determinable in conducted assays
n.t. not tested, – no inhibitory effects observed up to 67 µg/mL.

Table S3. NMR chemical shifts (^{1}H 500 MHz, ^{13}C 125 MHz) of Fulvoferruginin (A) (**1**) in methanol-d_4.

Pos.	δ_C	mult	δ_H	mult (J, Hz)
1	143.6	CH	6.11	d (11.2)
2	126.6	CH	5.61	d (11.2)
3	133.9	C		
4	125.3	CH	5.70	dd (2.2, 1.14)
5	78.6	CH	4.63	dt (12.6, 2.2)
6	57.6	CH	2.40	d (12.6)
7	45.3	C		
8	38.4	CH_2	2.10	td (13.6, 12.0, 6.5)
8			1.74	dd (12.0, 6.5)
9	36.2	CH_2	2.40	dt (13.6, 6.6)
9			1.95	dd (13.6, 6.6)
10	80.7	C		
11	144.9	C		
12	27.9	CH_3	1.91	t (1.9)
13	19.8	CH_3	0.89	s
14	121.7	CH_2	5.96	s
14			5.73	s
15	171.8	C		

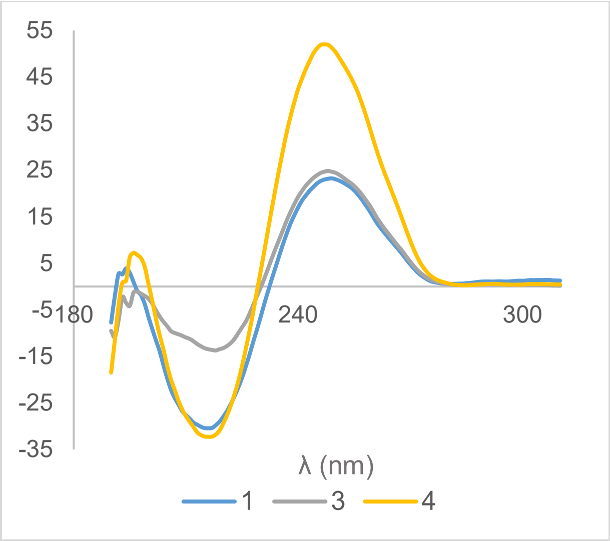

Figure S1. ECD spectra of Fulvoferruginin (**1**), Fulvoferruginin C (**3**) and D (**4**) in methanol (Δ_ε [cm^2/mmol],*c* 1.0). λ_{max} ($\Delta\varepsilon$) for **1**: 216 (-30.4), 249 (23.2); for **3**: 218 (-13.7), 248 (24.8); for **4**: 216 (-32.3), 247 (52.0).

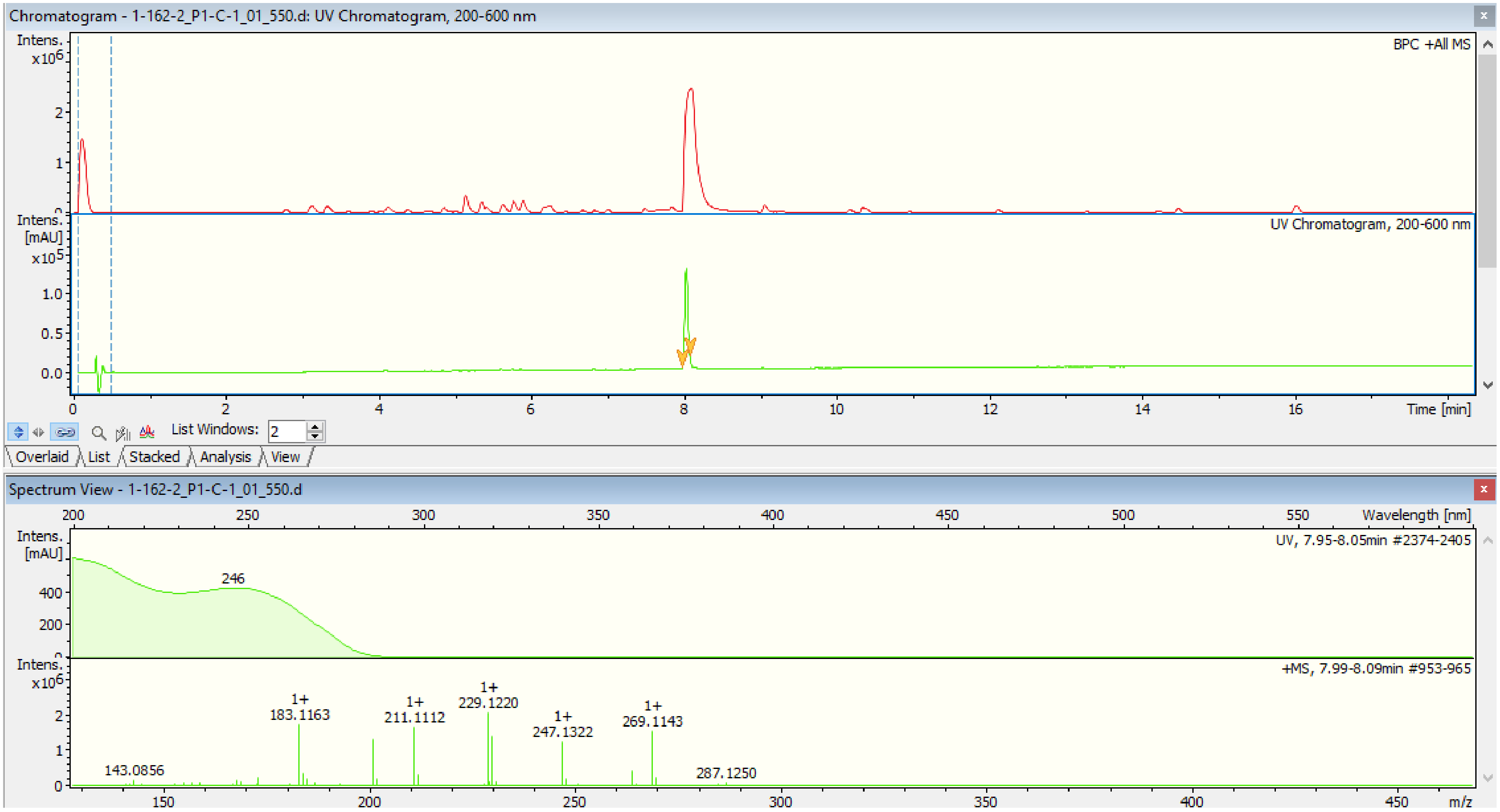

HRESIMS of Fulvoferruginin A (**1**).

^{1}H NMR spectrum (500 MHz, methanol-d_4) of Fulvoferruginin (A) (**1**).

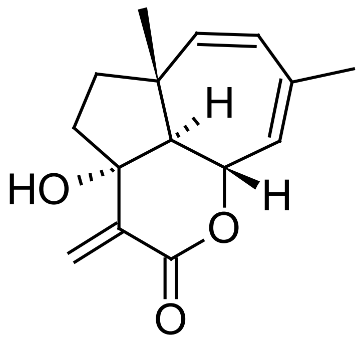

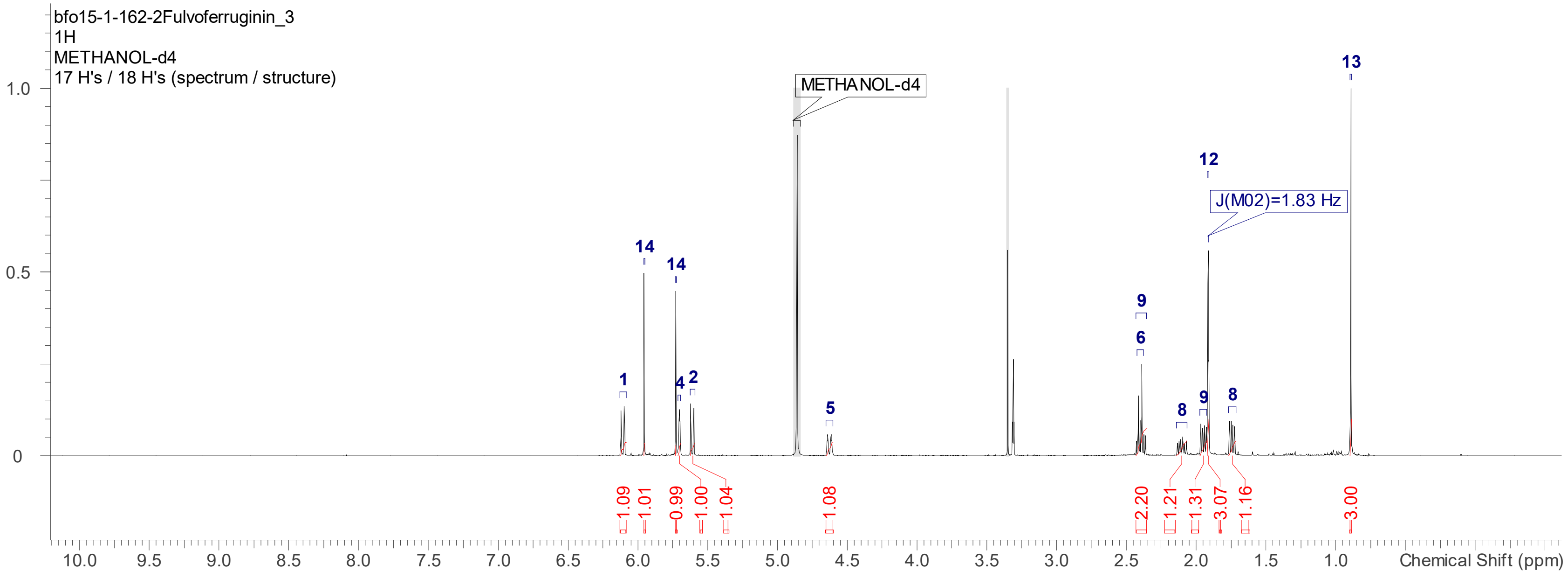

^{13}C NMR spectrum (125 MHz, methanol-d_4) of Fulvoferruginin A (**1**).

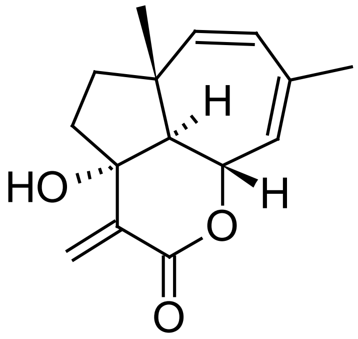

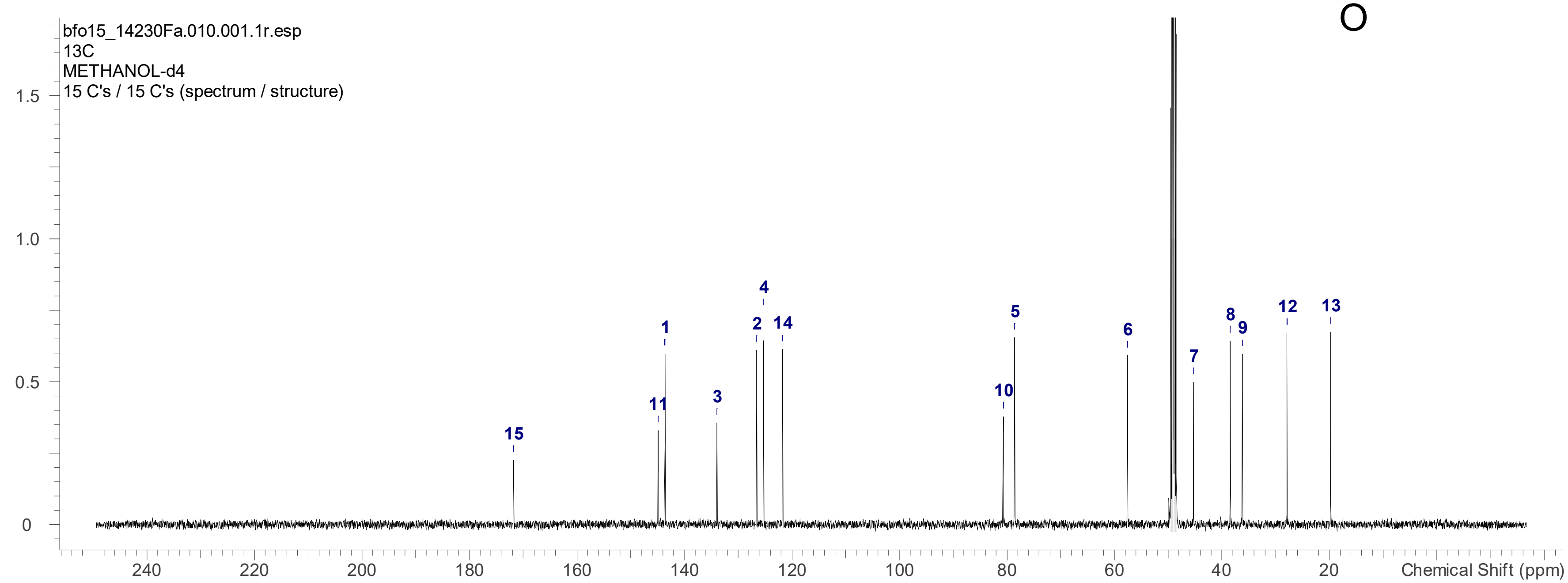

HSQC-dept NMR spectrum (500 MHz, methanol-d_4) of Fulvoferruginin A (**1**).

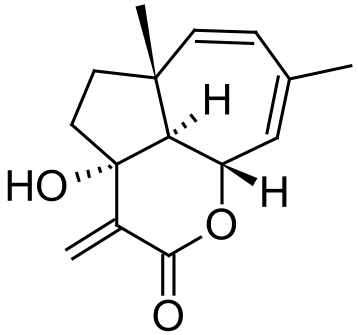

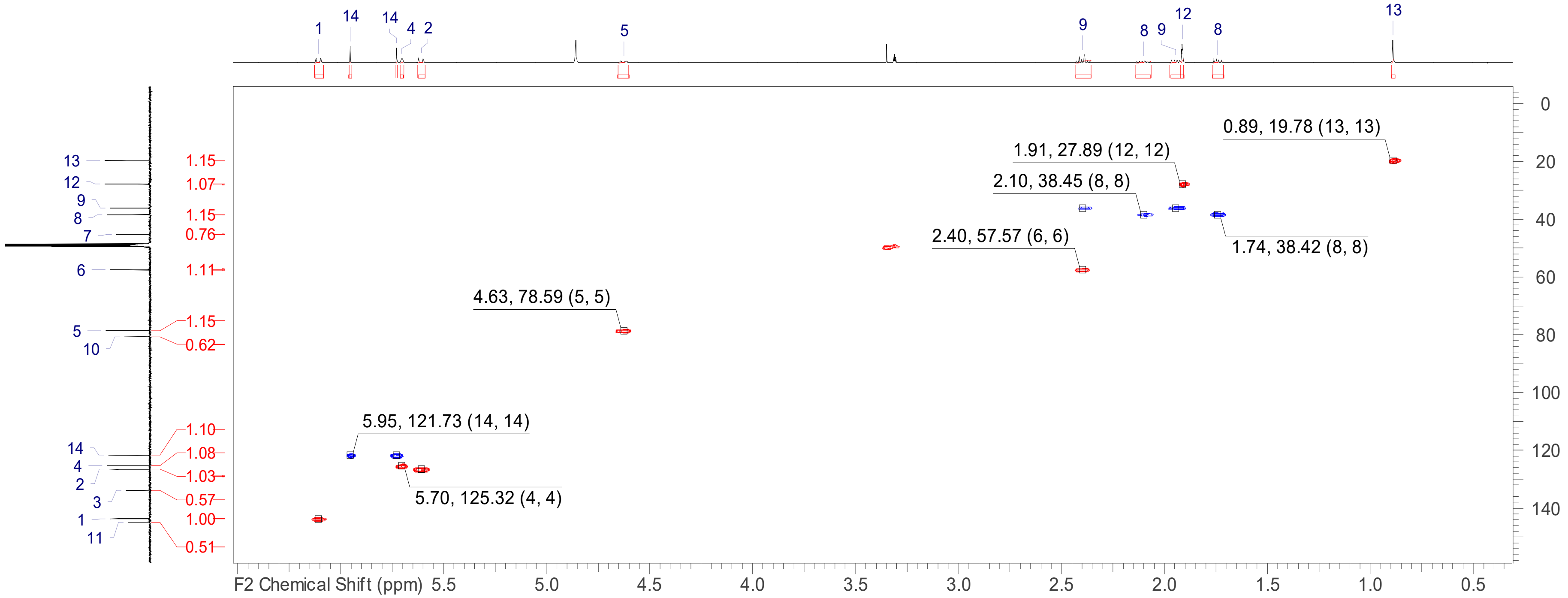

HMBC NMR spectrum (500 MHz, methanol-*d*4) of Fulvoferruginin A (**1**).

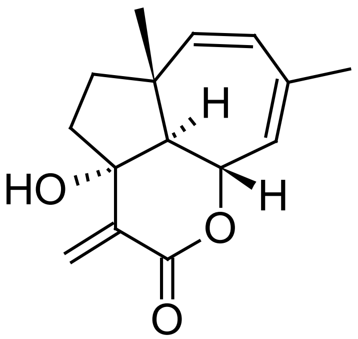

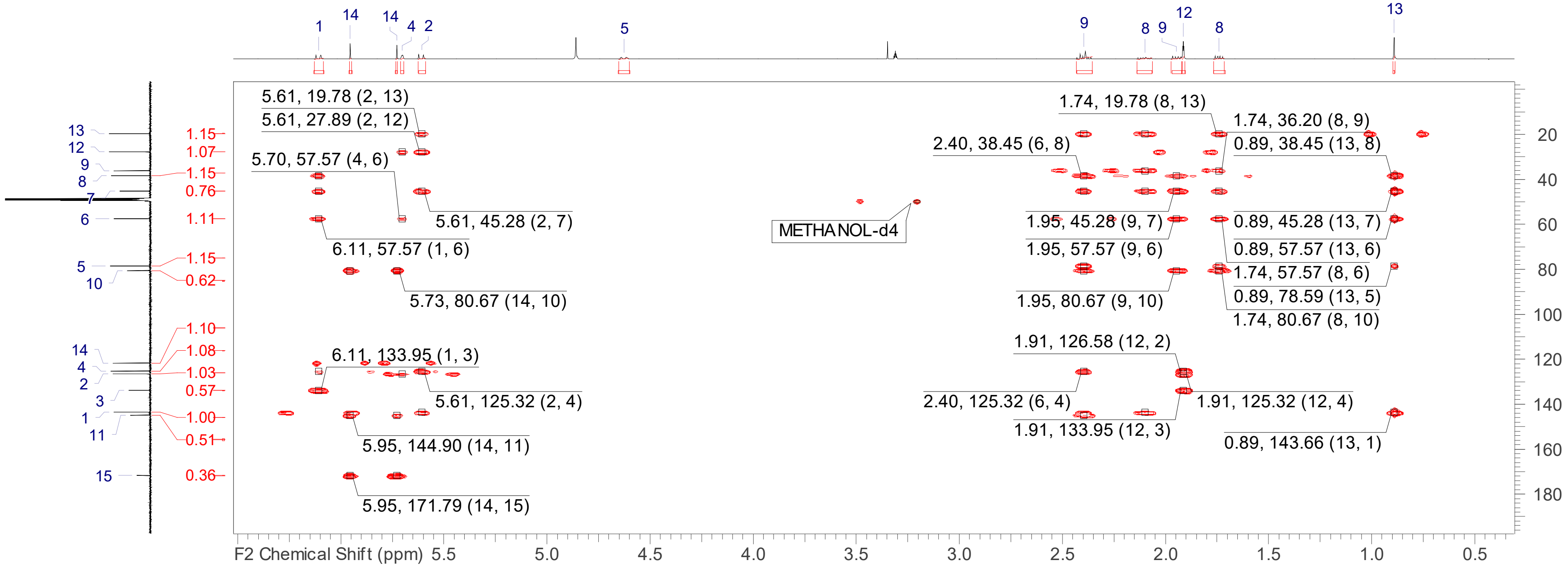

COSY NMR spectrum (500 MHz, methanol-d_4) of Fulvoferruginin A (**1**).

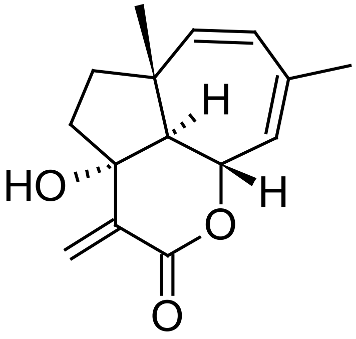

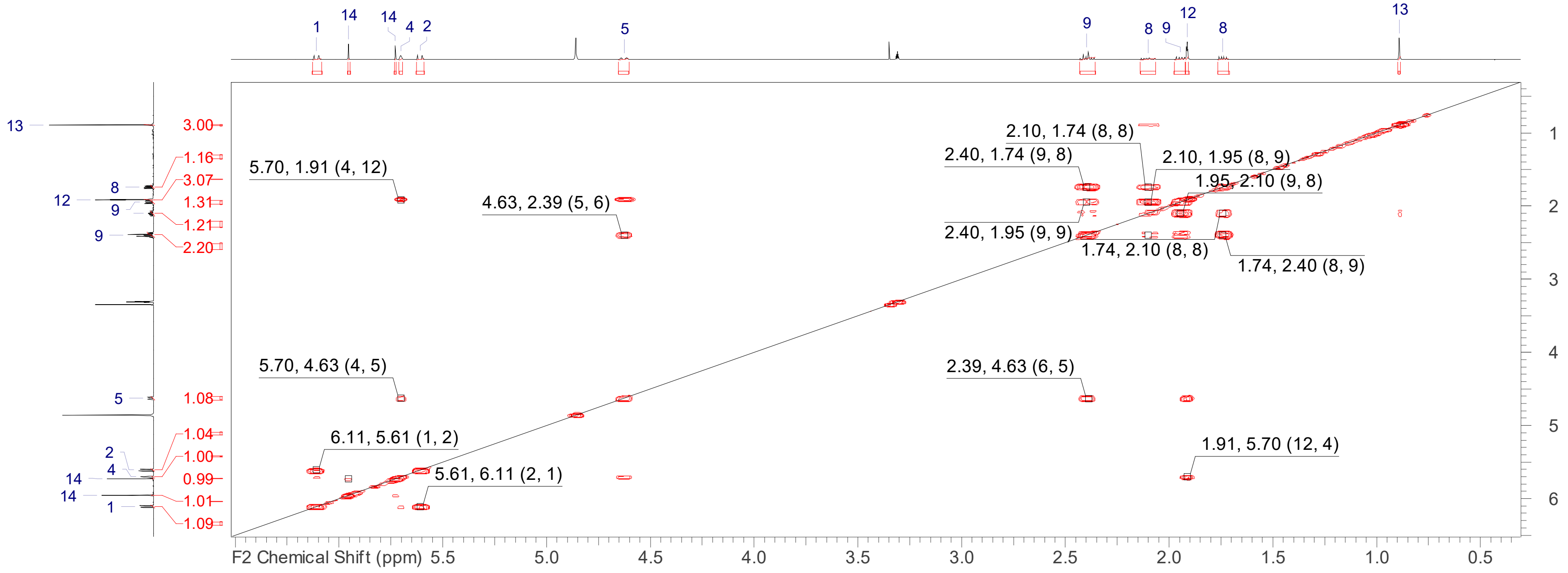

ROESY NMR spectrum (500 MHz, methanol-d_4) of Fulvoferruginin A (**1**).

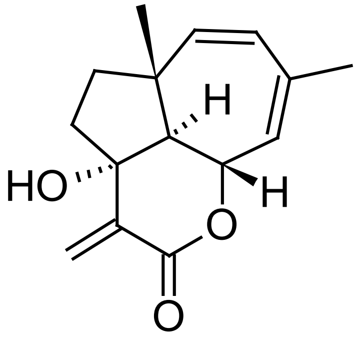

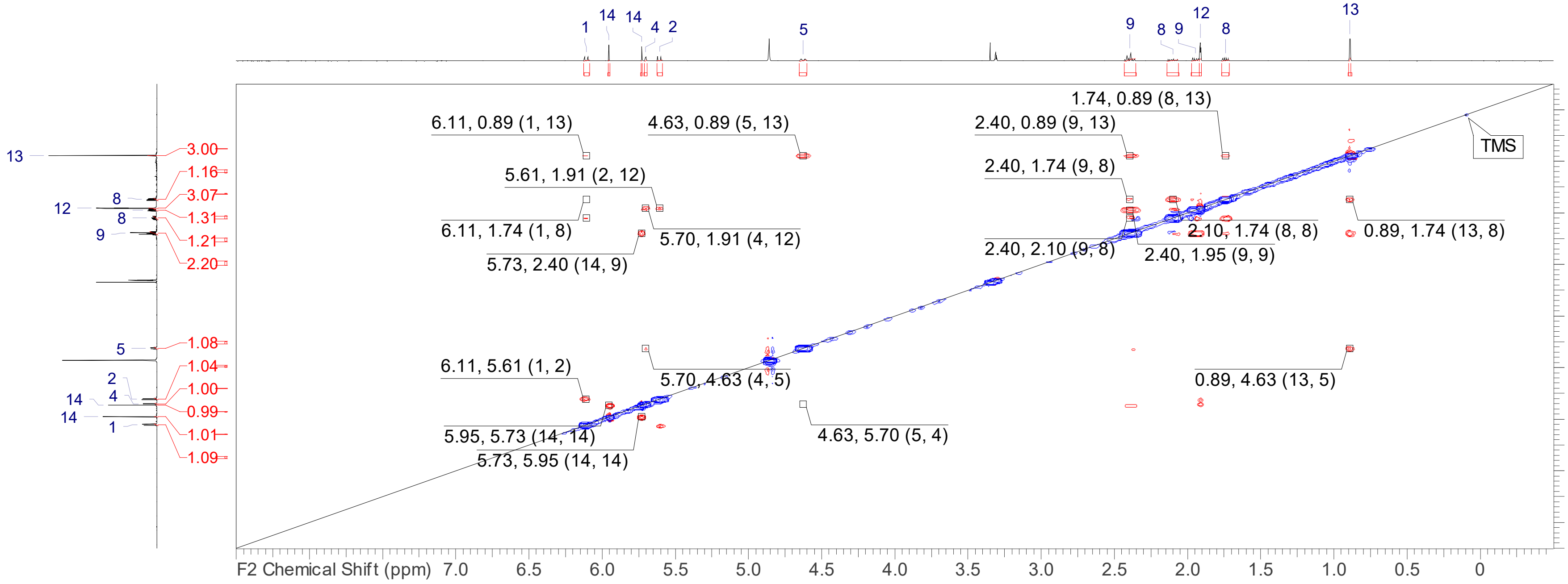

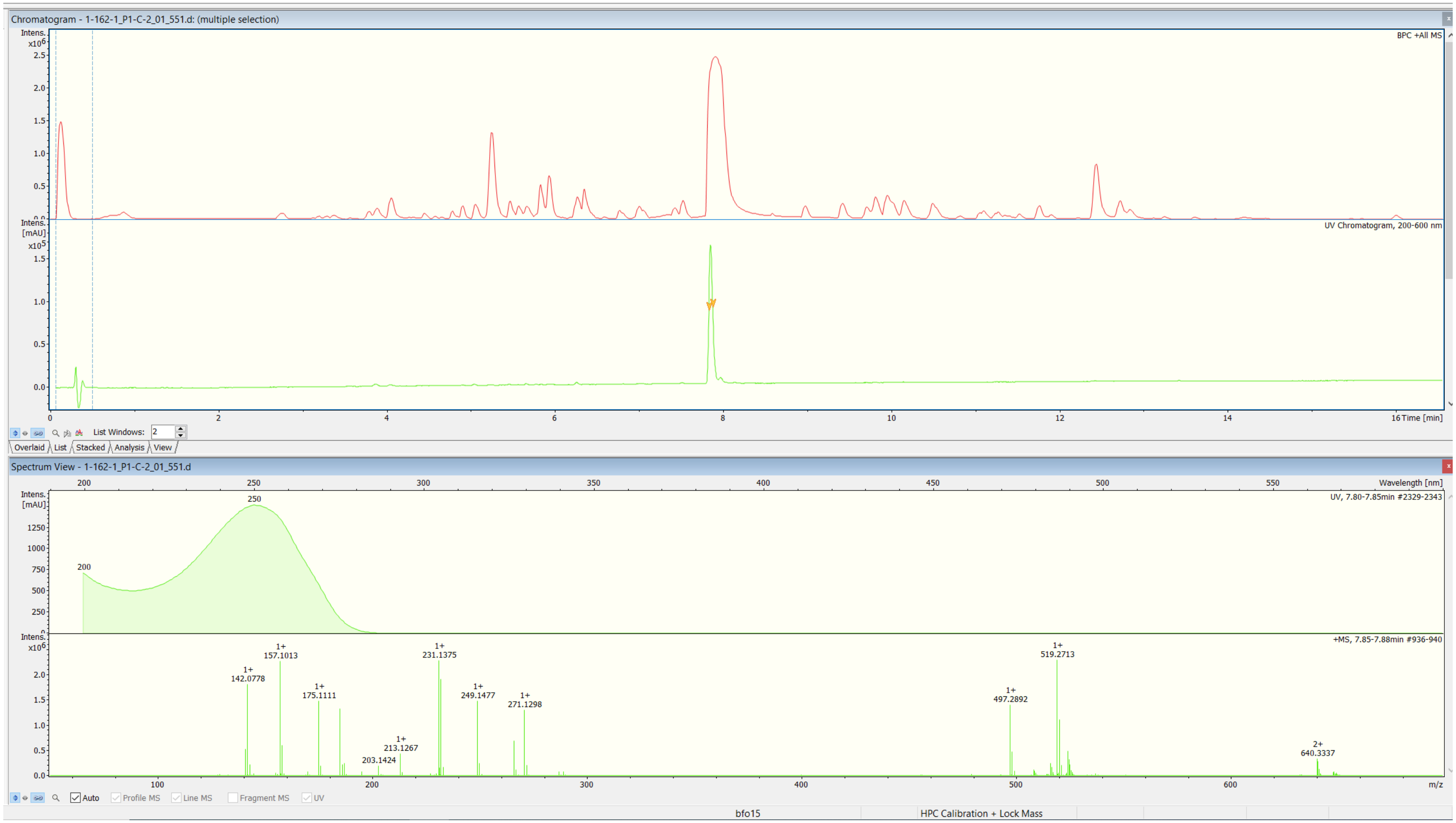

HRESIMS of Fulvoferruginin B (**2**).

^{1}H NMR spectrum (700 MHz, methanol-*d*4) of Fulvoferruginin B (**2**).

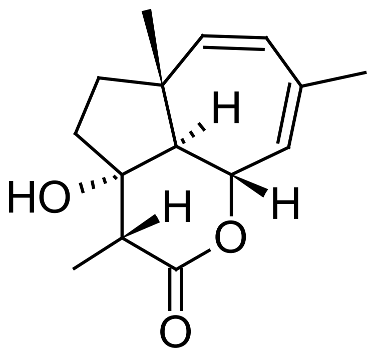

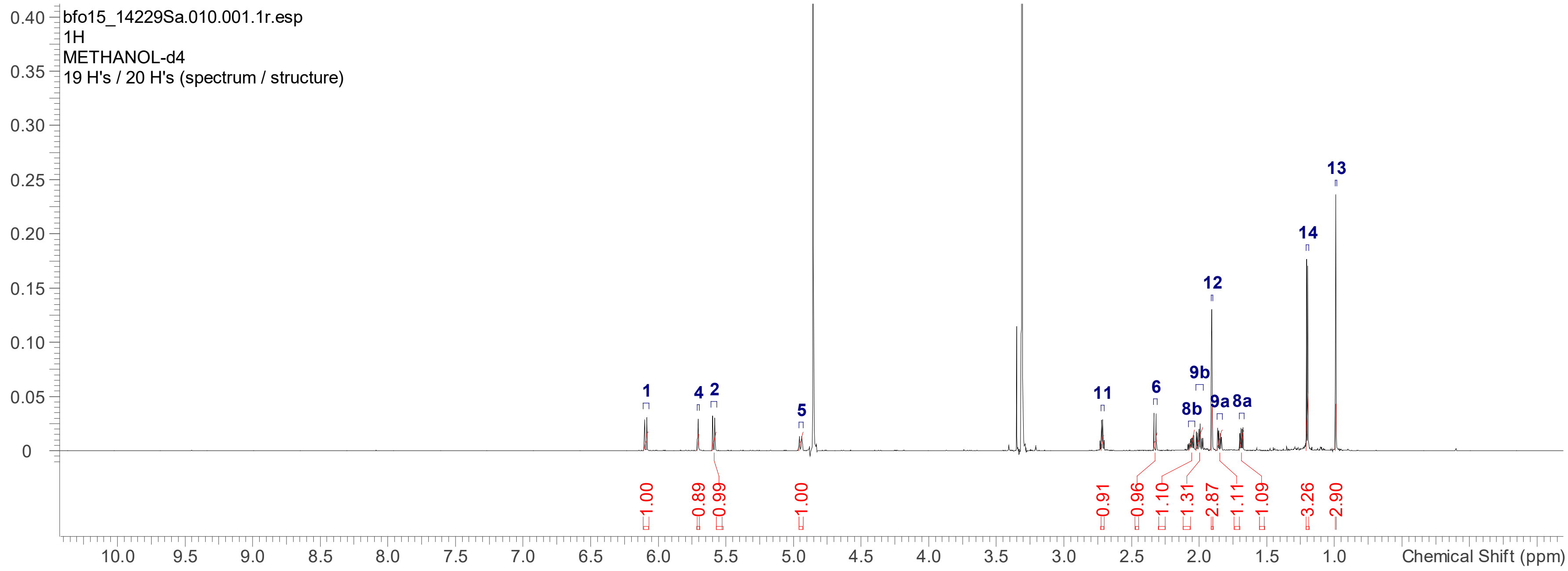

^{13}C NMR spectrum (176 MHz, methanol-d_4) of Fulvoferruginin B (**2**).

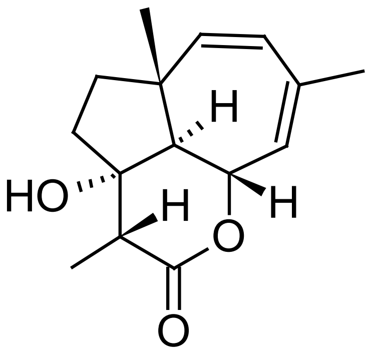

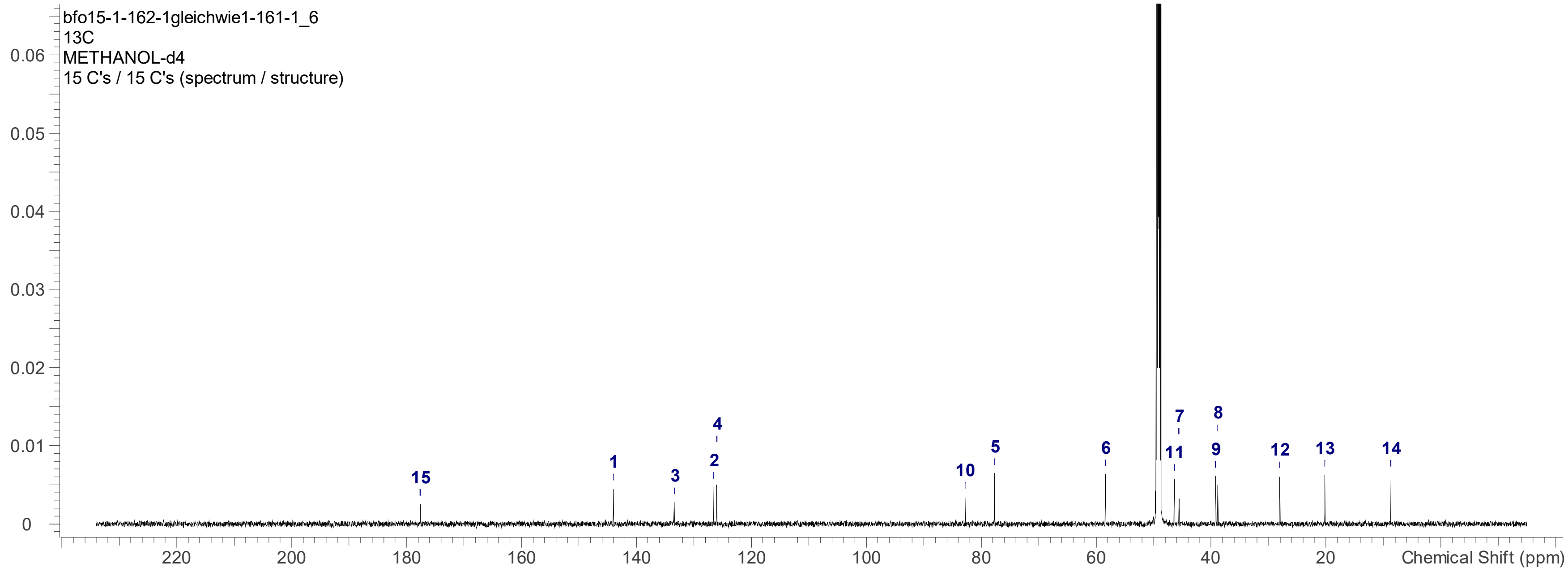

HSQC-dept NMR spectrum (700 MHz, methanol-d_4) of Fulvoferruginin B (**2**).

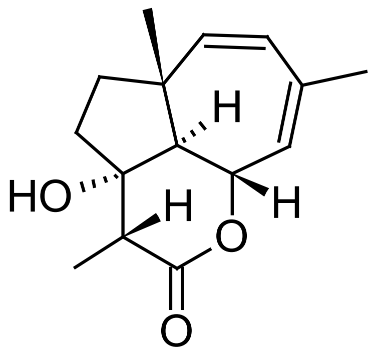

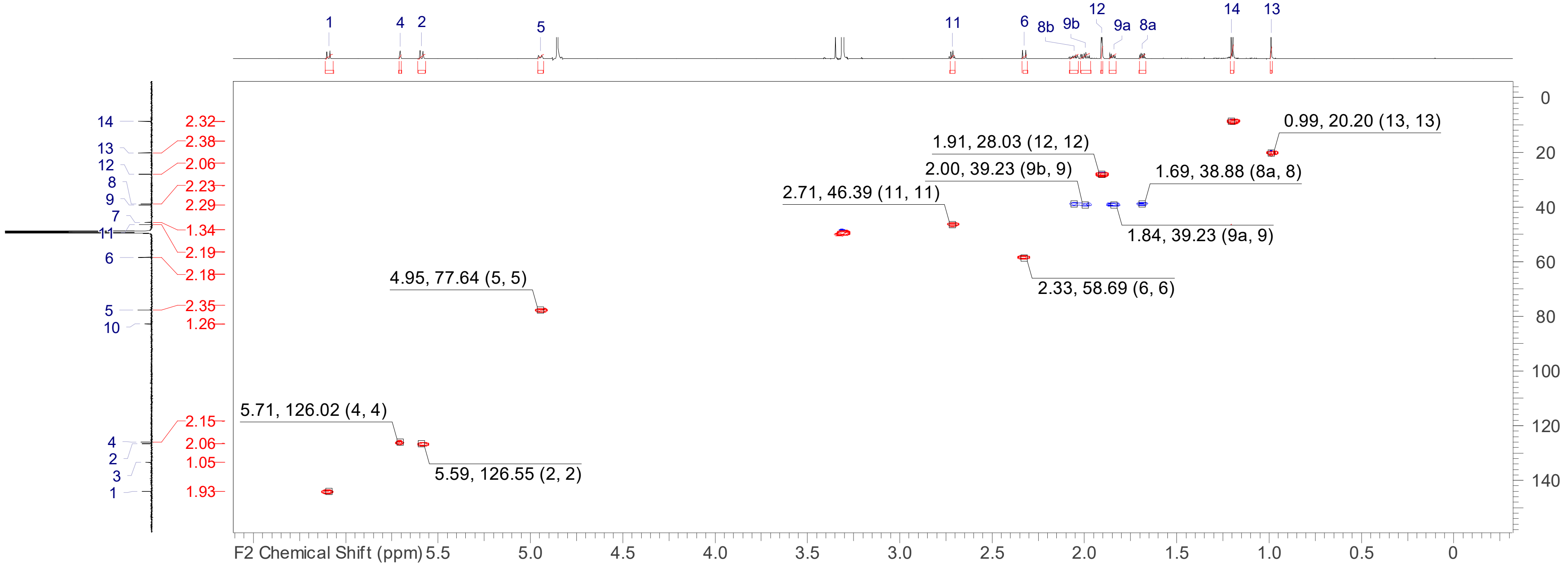

HMBC NMR spectrum (700 MHz, methanol-d_4) of Fulvoferruginin B (**2**).

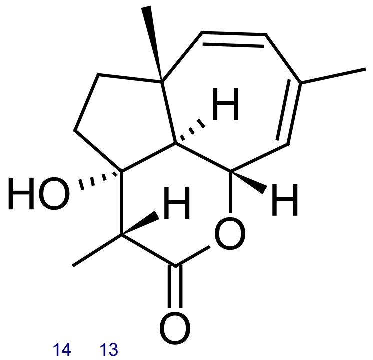

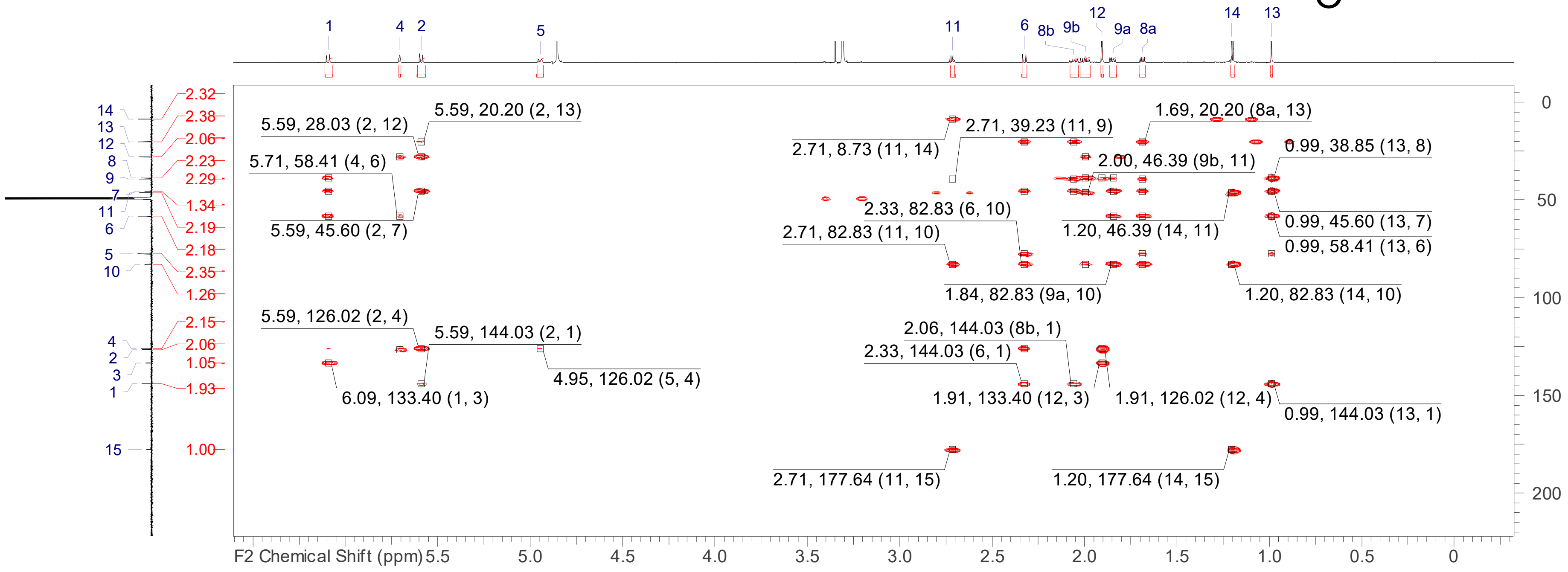

COSY NMR spectrum (700 MHz, methanol-d_4) of Fulvoferruginin B (**2**).

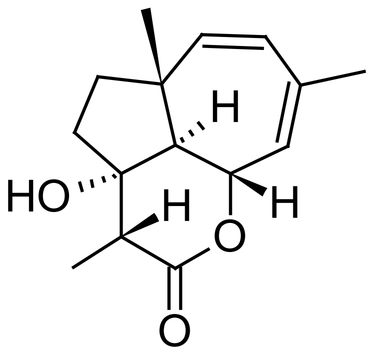

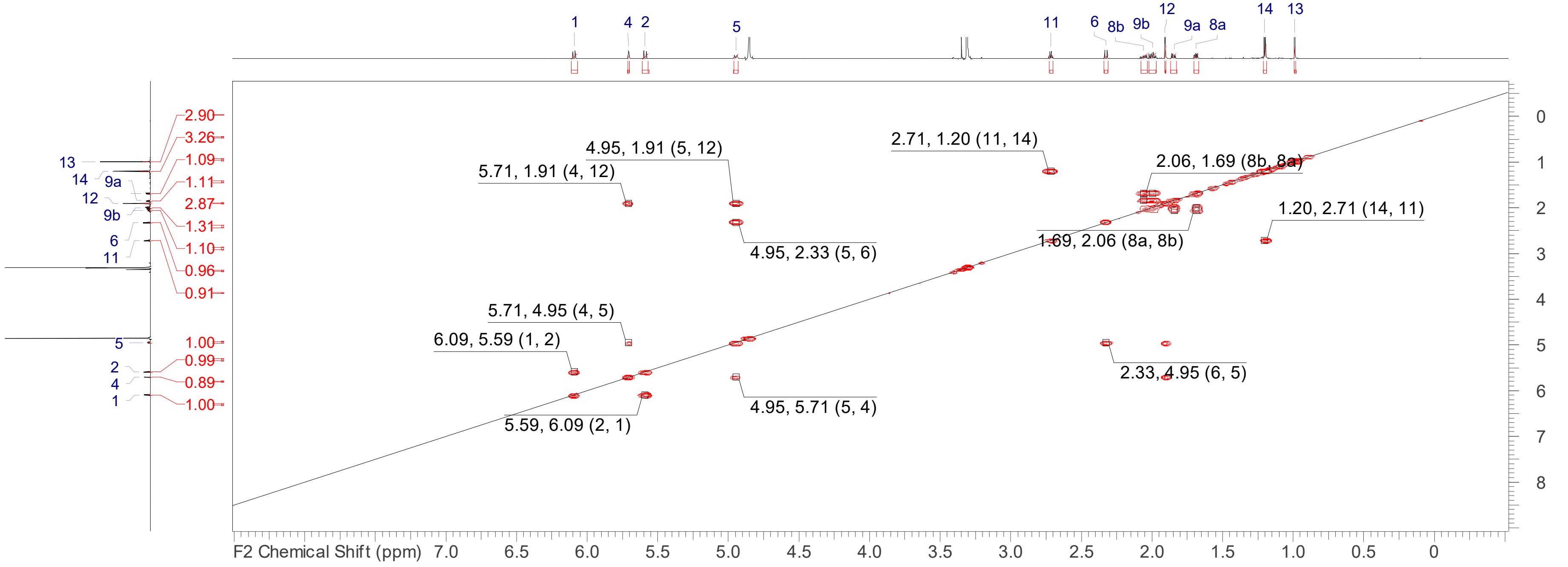

ROESY NMR spectrum (700 MHz, methanol-d_4) of Fulvoferruginin B (**2**).

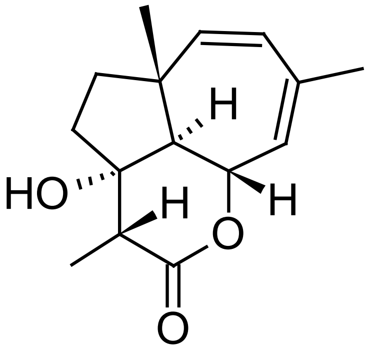

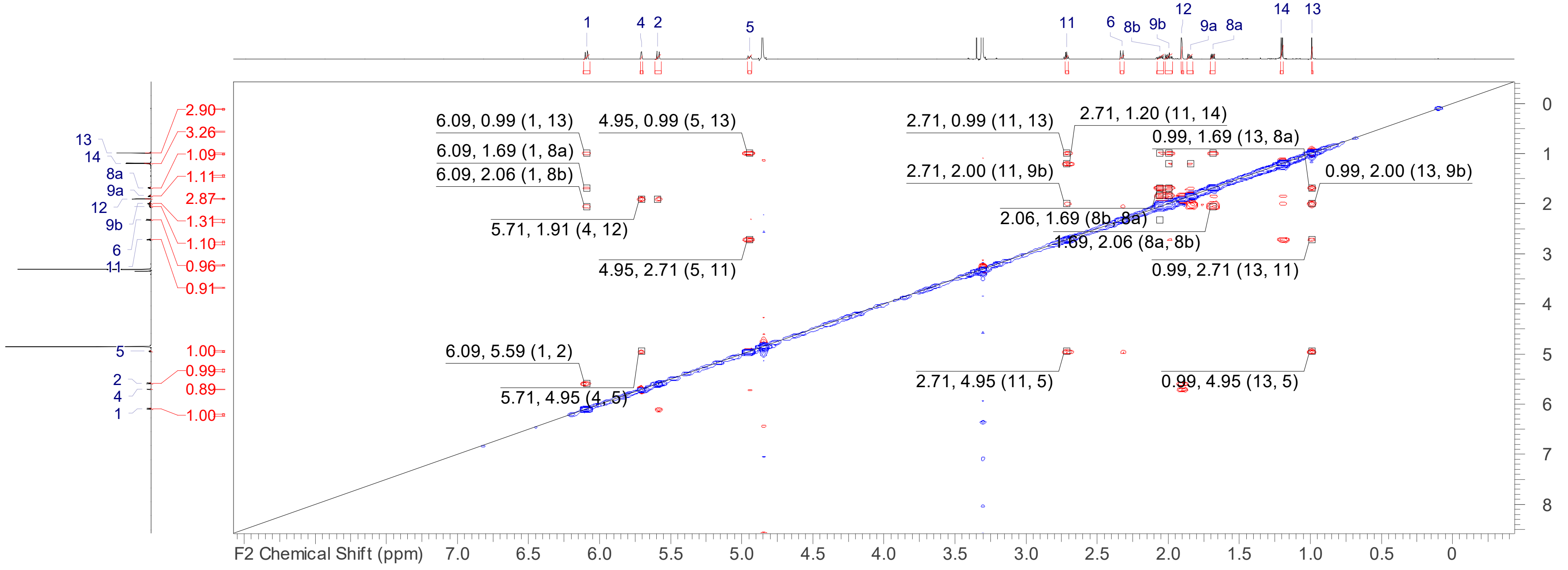

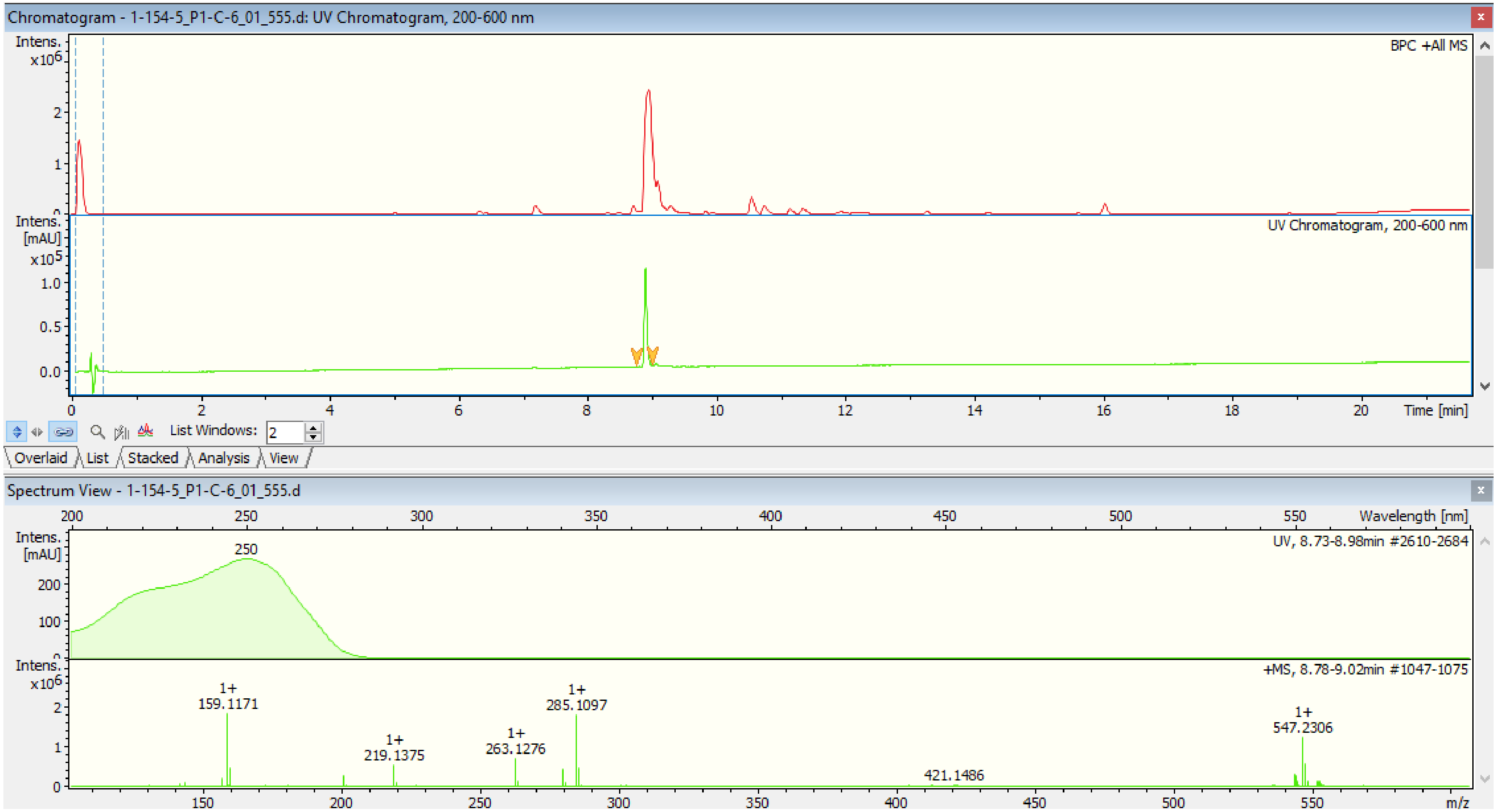

HRESIMS of Fulvoferruginin C (**3**).

^{1}H NMR spectrum (700 MHz, methanol-*d*4) of Fulvoferruginin C (**3**).

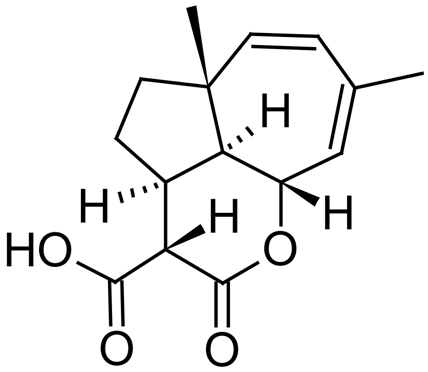

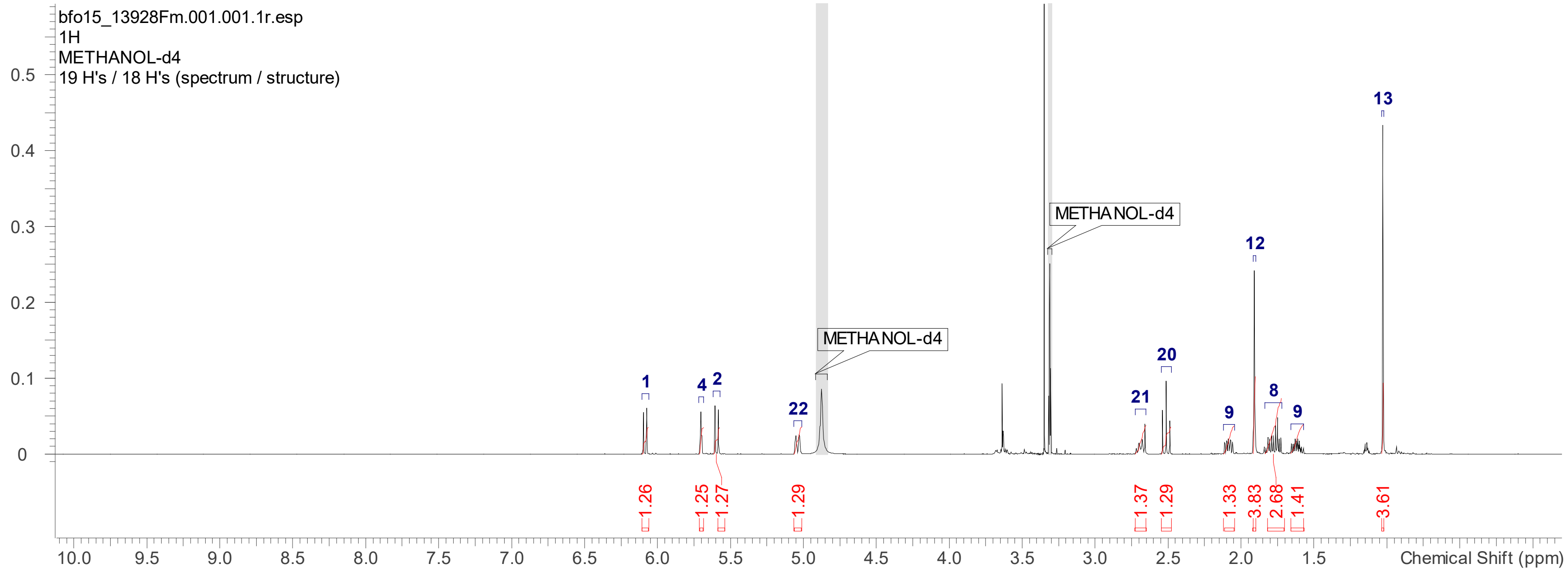

^{13}C NMR spectrum (176 MHz, methanol-d_4) of Fulvoferruginin C (**3**).

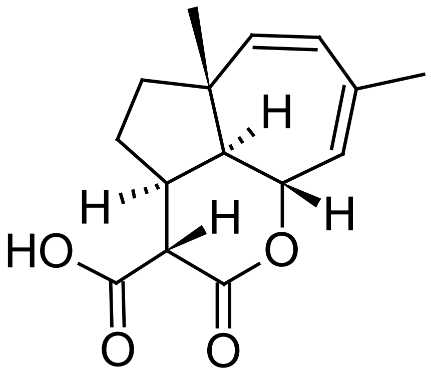

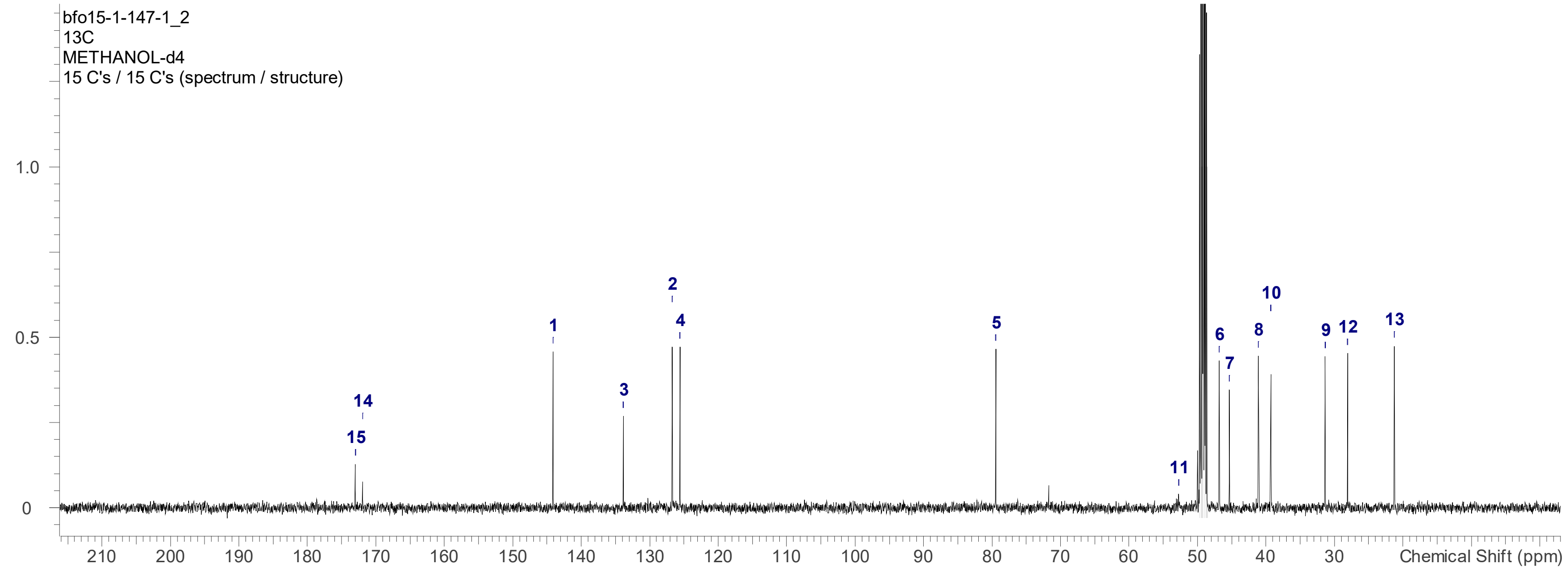

HSQC-dept NMR spectrum (700 MHz, methanol-d_4) of Fulvoferruginin C (**3**).

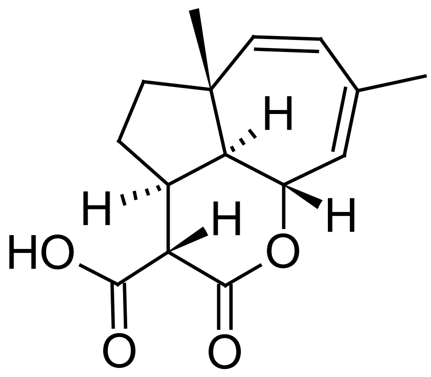

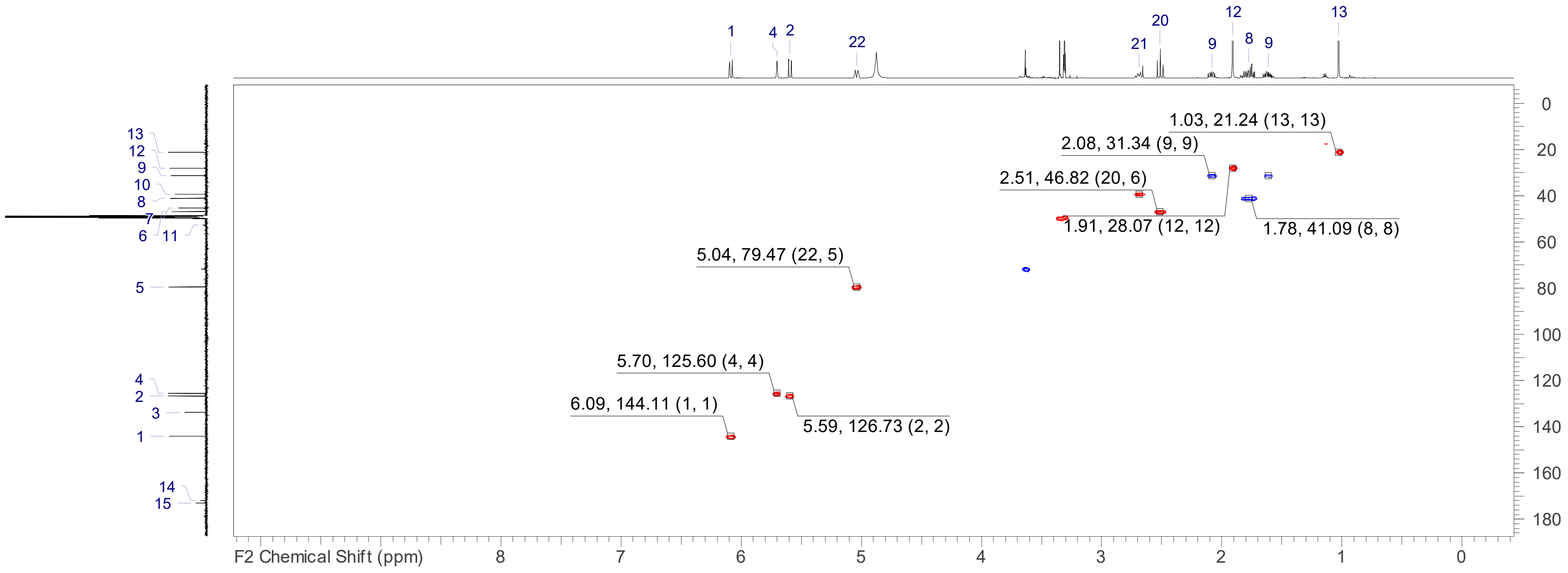

HMBC NMR spectrum (700 MHz, methanol-d_4) of Fulvoferruginin C (**3**).

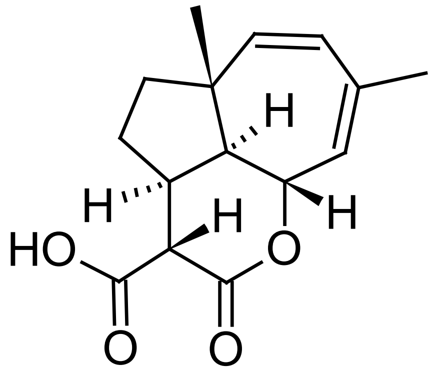

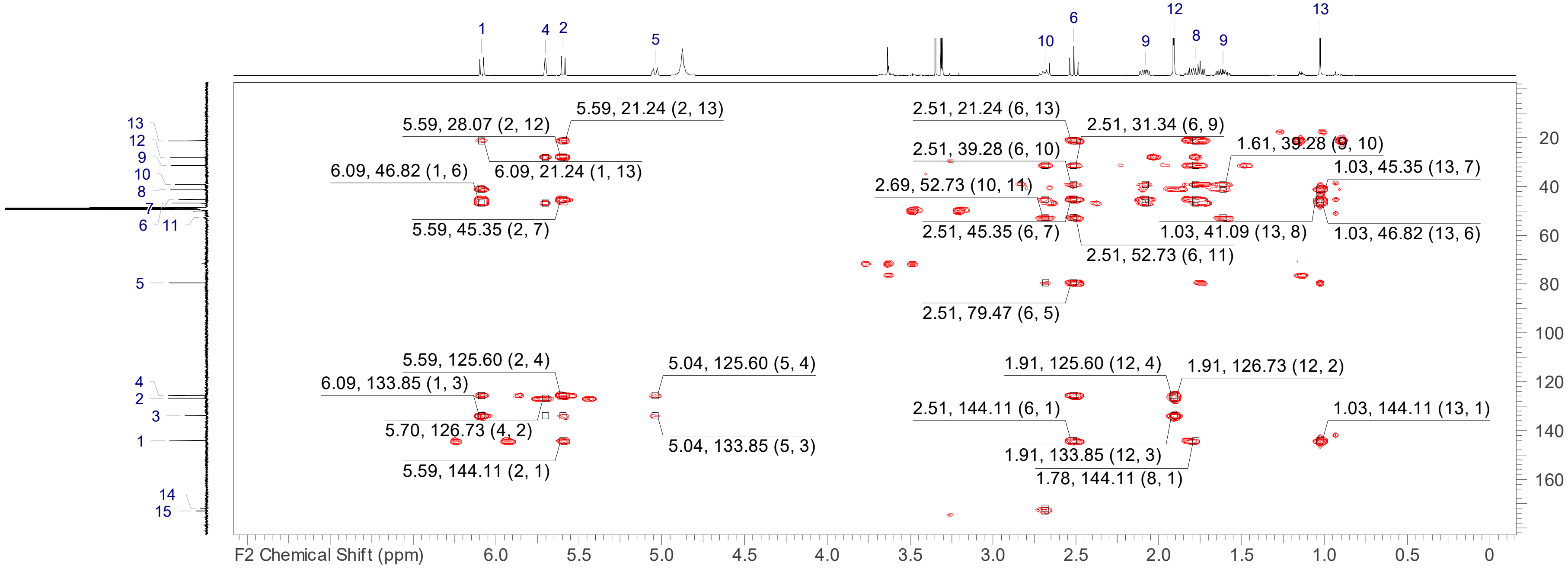

COSY NMR spectrum (700 MHz, methanol-*d*4) of Fulvoferruginin C (**3**).

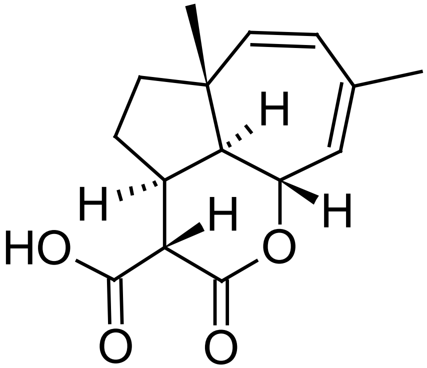

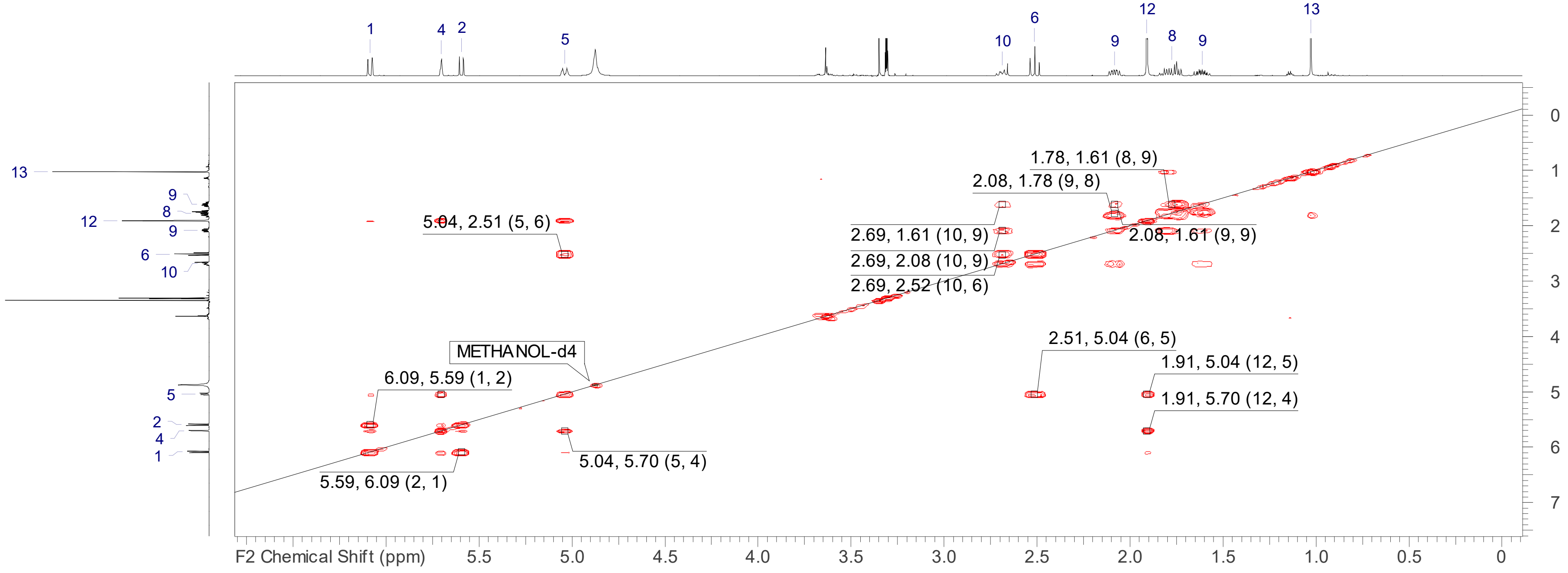

ROESY NMR spectrum (700 MHz, methanol-d_4) of Fulvoferruginin C (**3**).

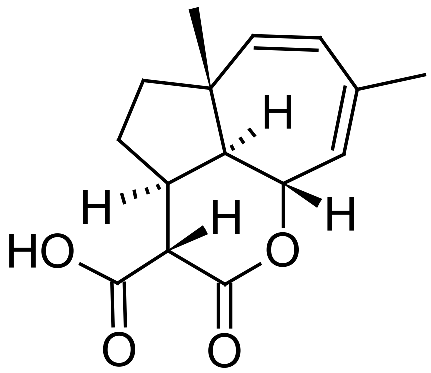

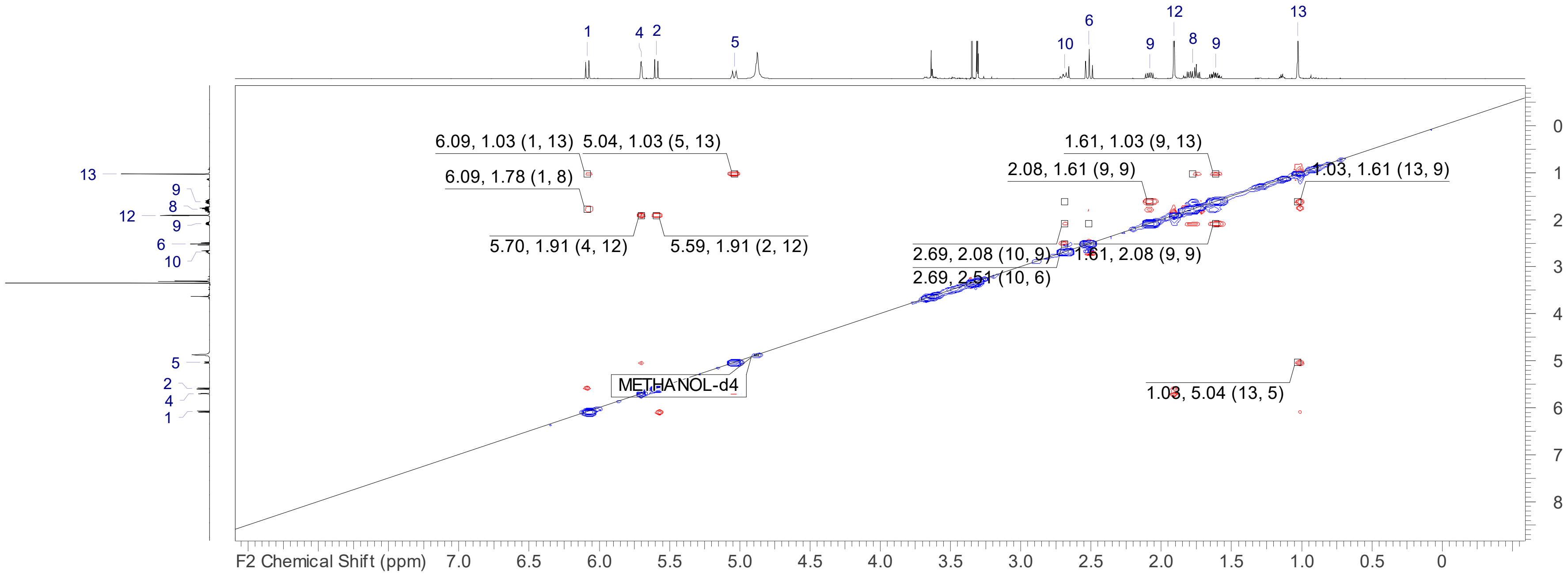

^{1}H NMR spectrum (700 MHz, chloroform-*d*) of Fulvoferruginin C (**3**).

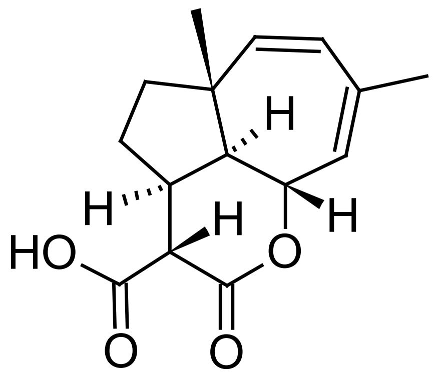

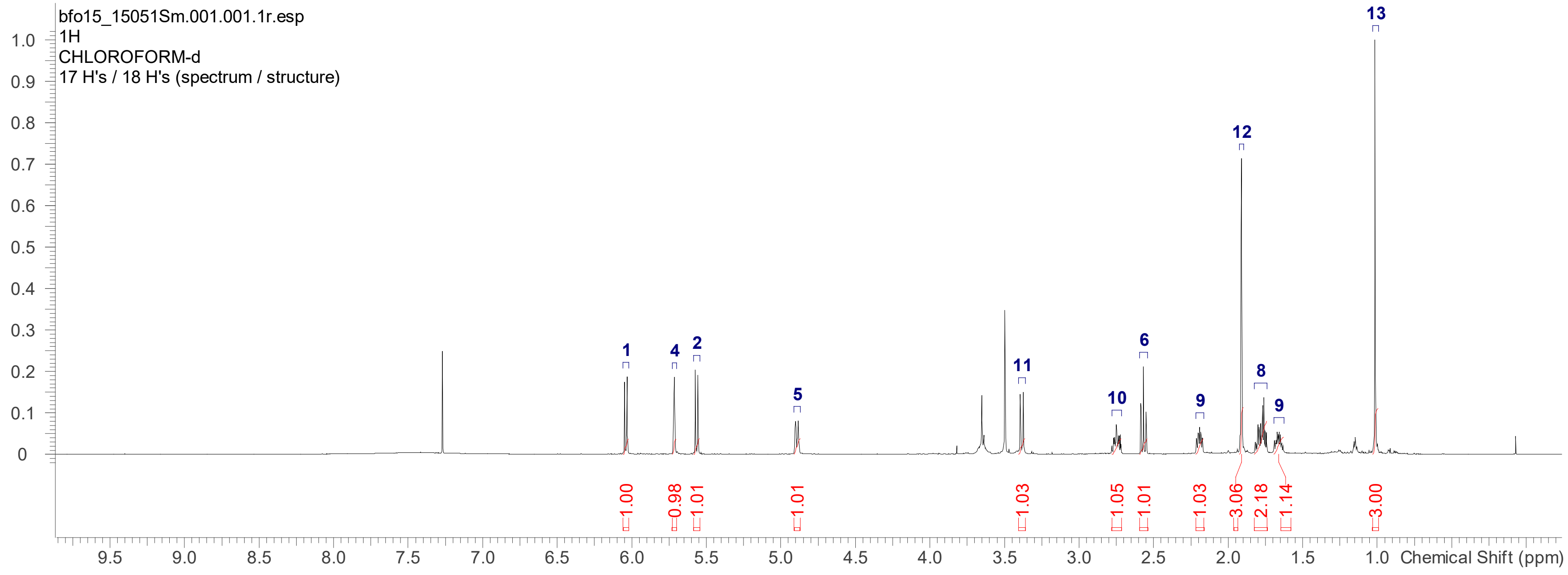

^{13}C NMR spectrum (176 MHz, chloroform-*d*) of Fulvoferruginin C (**3**).

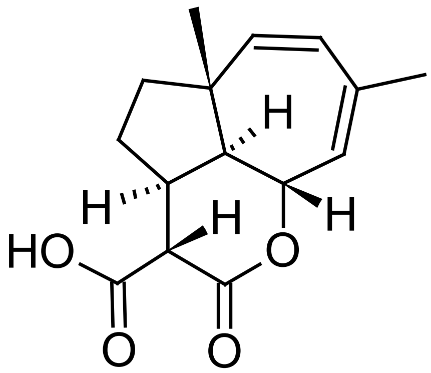

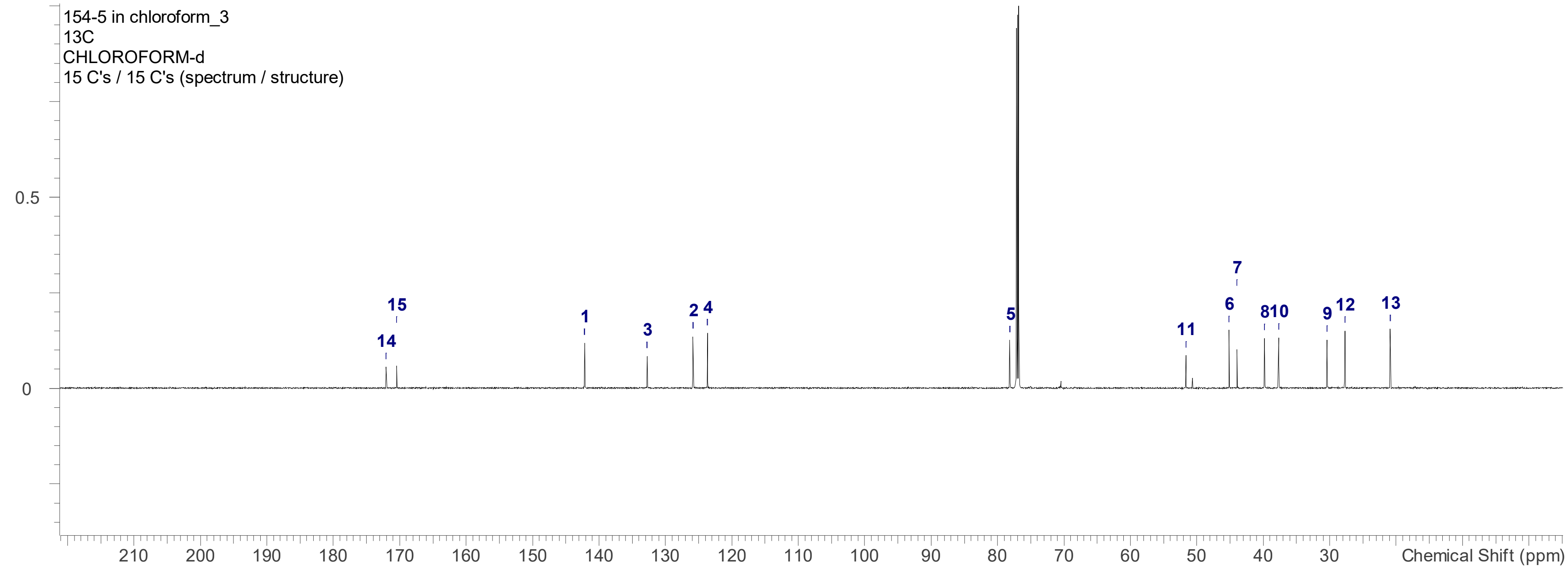

HSQC-dept NMR spectrum (700 MHz, chloroform-*d*) of Fulvoferruginin C (**3**).

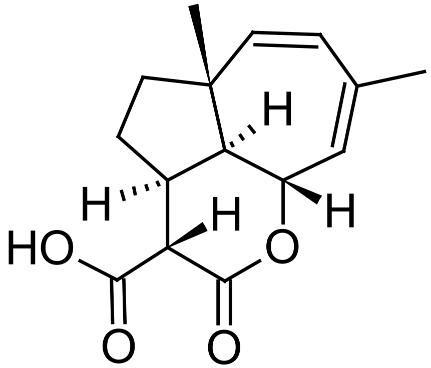

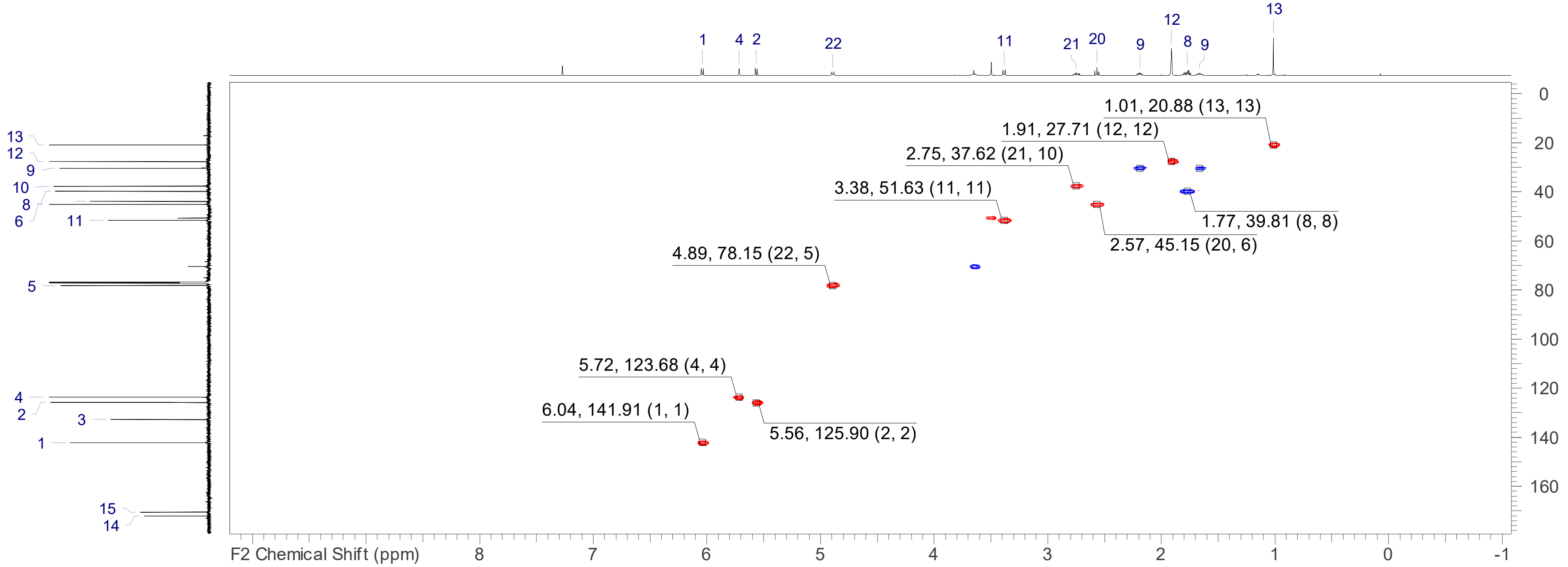

HMBC NMR spectrum (700 MHz, chloroform-*d*) of Fulvoferruginin C (**3**).

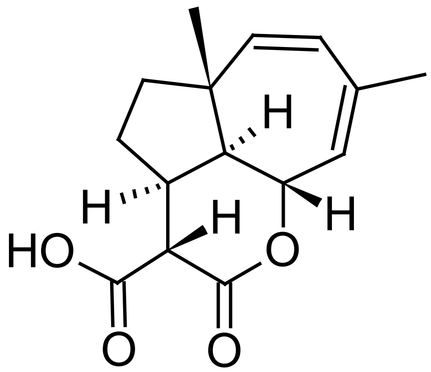

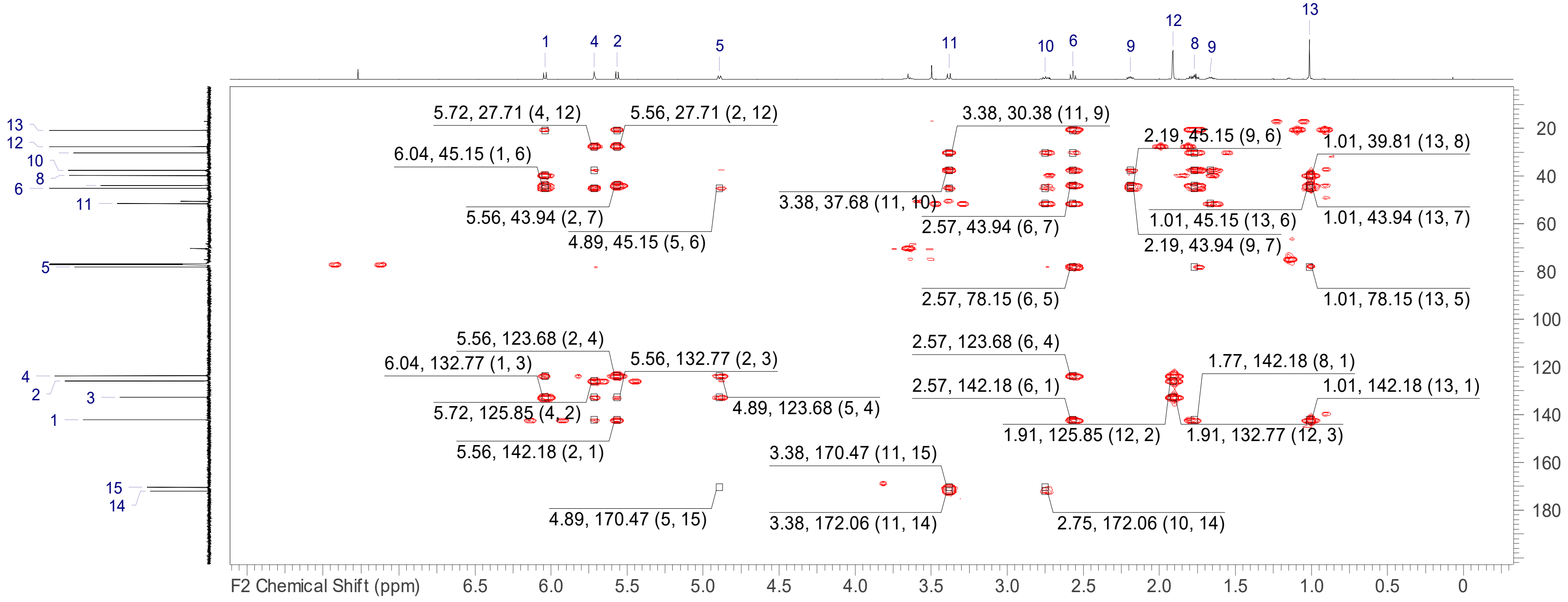

COSY NMR spectrum (700 MHz, chloroform-*d*) of Fulvoferruginin C (**3**).

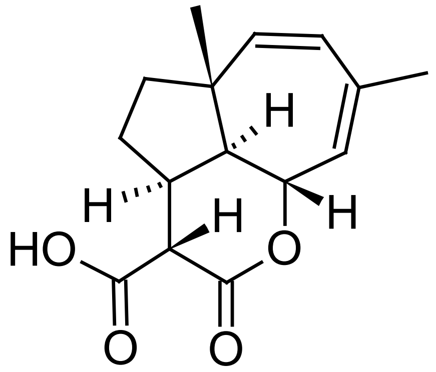

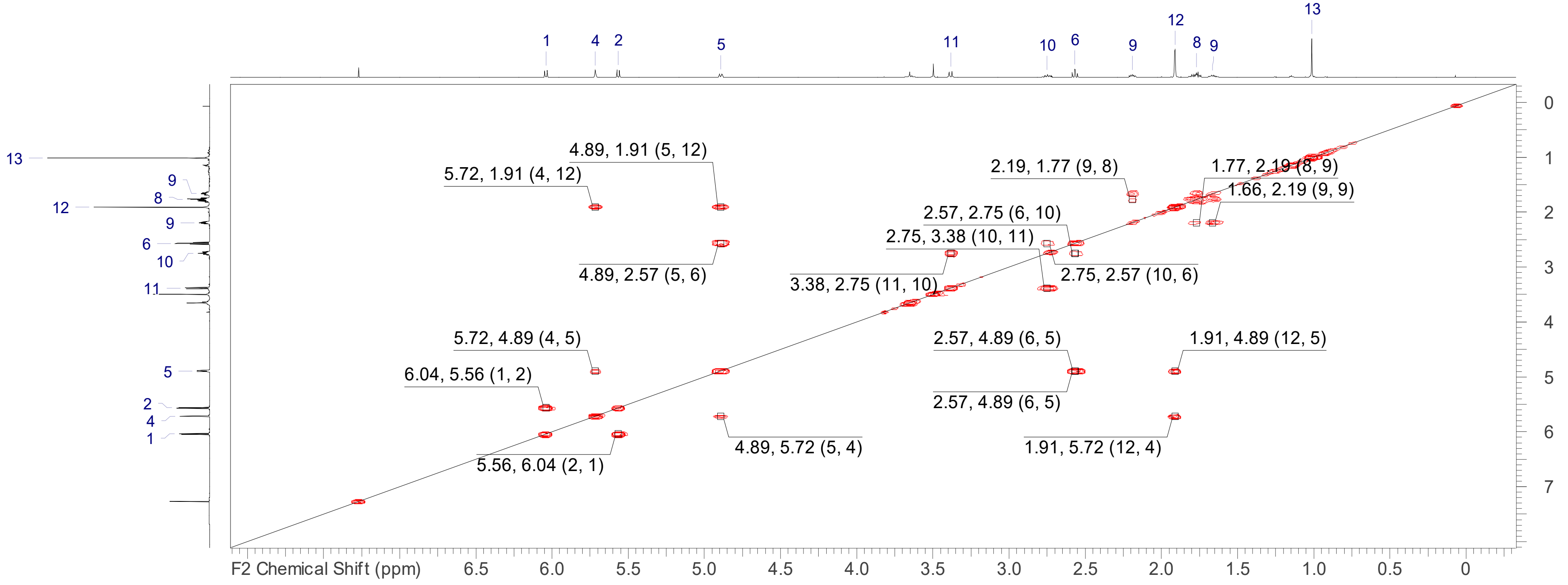

ROESY NMR spectrum (700 MHz, chloroform-*d*) of Fulvoferruginin C (**3**).

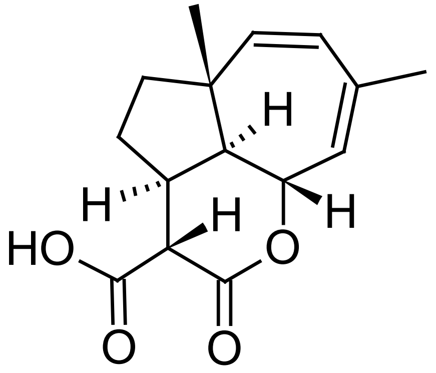

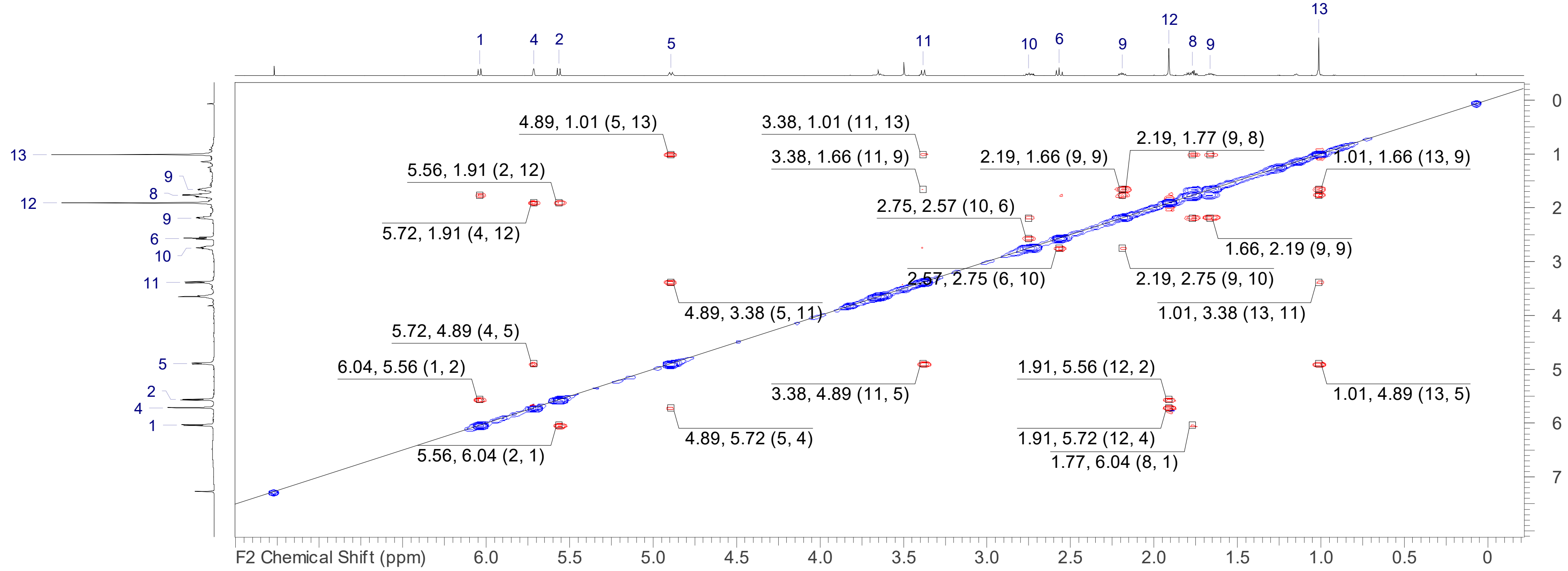

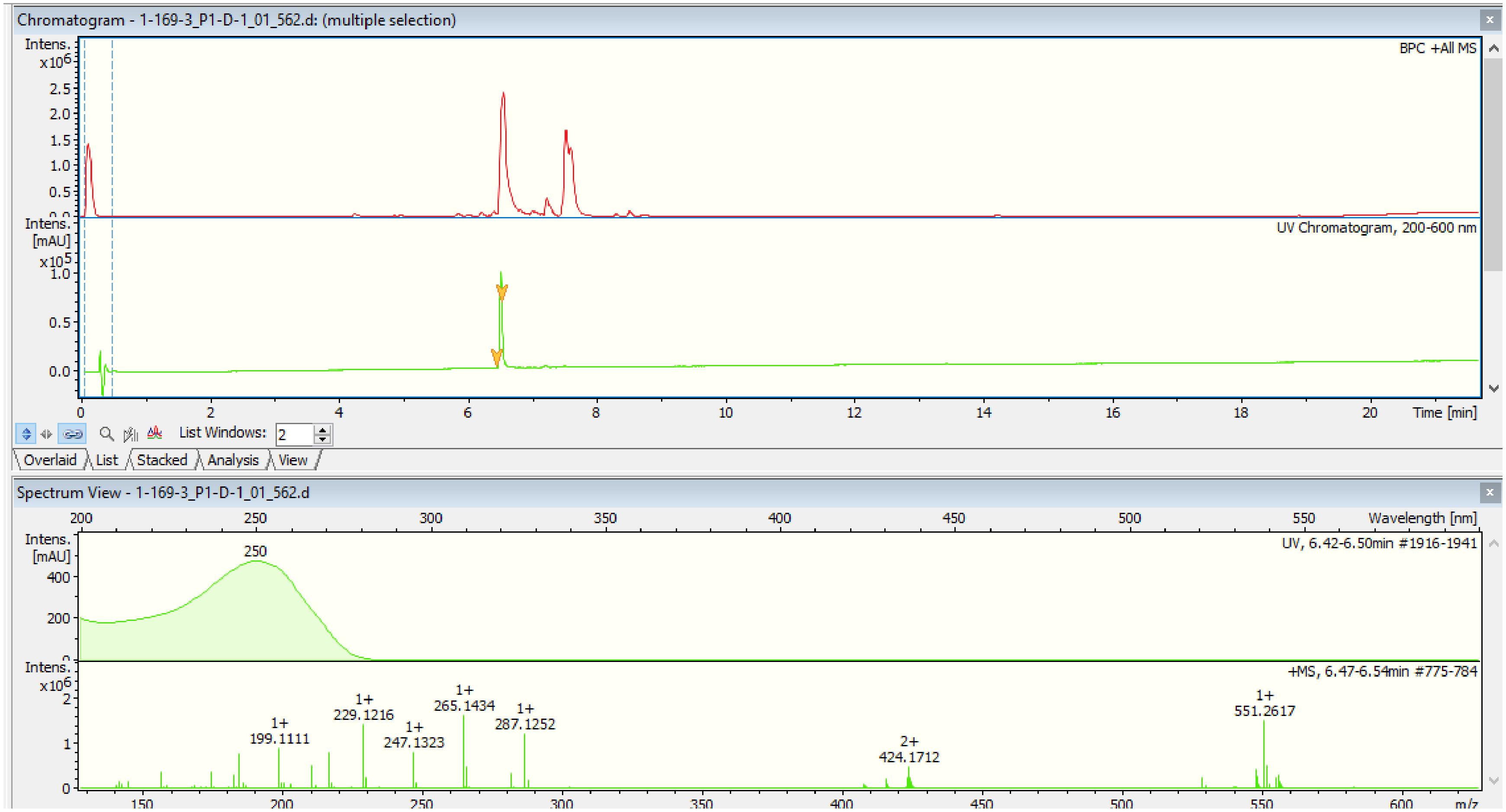

HRESIMS of Fulvoferruginin D (**4**).

^{1}H NMR spectrum (700 MHz, methanol-d_4) of Fulvoferruginin D (**4**).

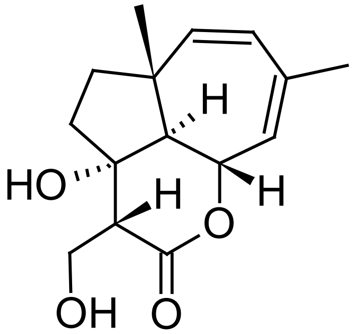

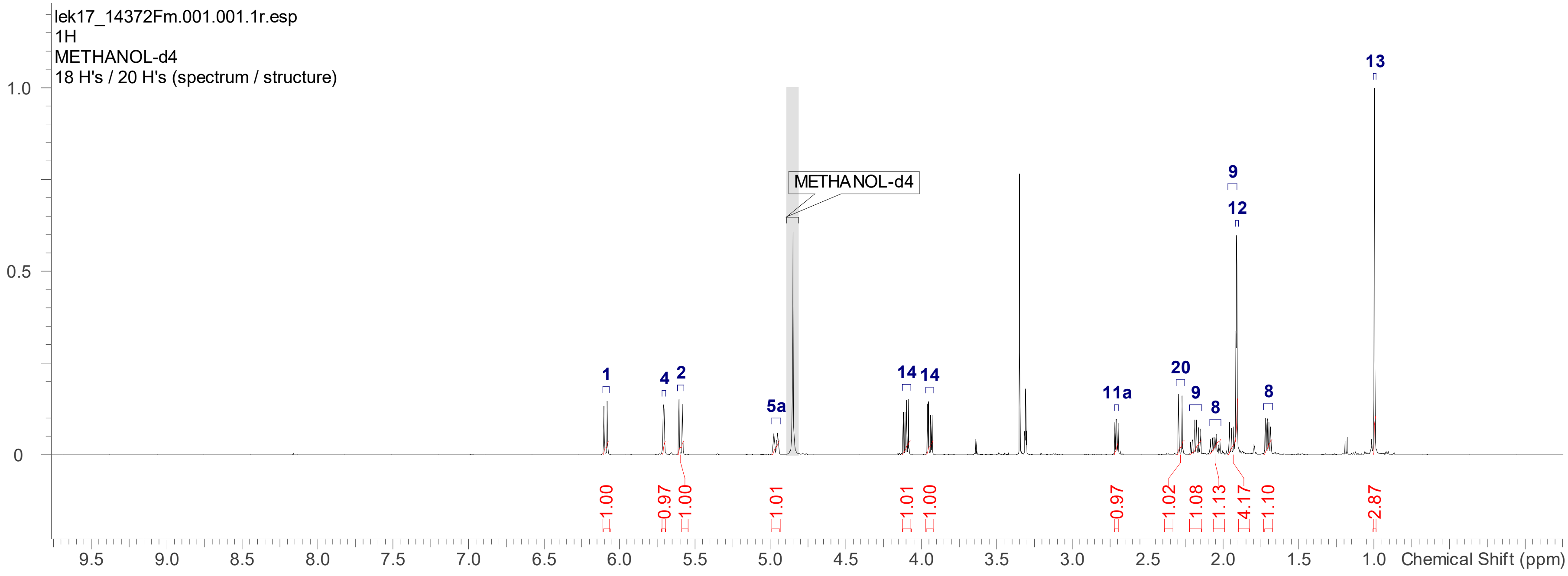

^{13}C NMR spectrum (176 MHz, methanol-d_4) of Fulvoferruginin D (**4**).

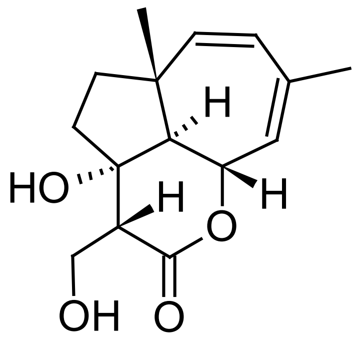

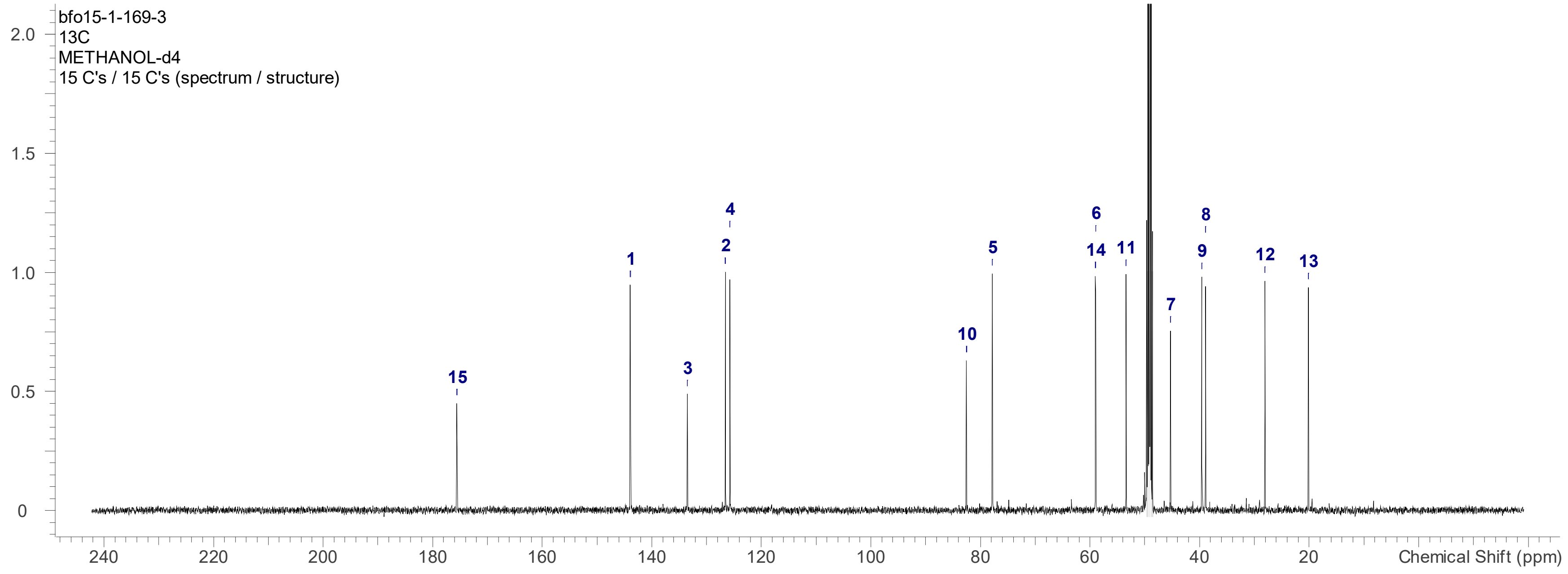

HSQC-dept NMR spectrum (700 MHz, methanol-d_4) of Fulvoferruginin D (**4**).

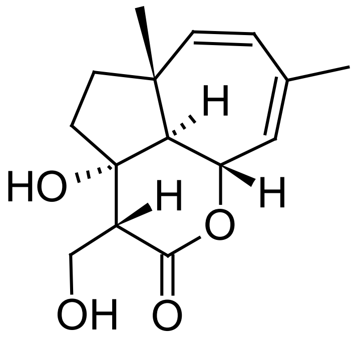

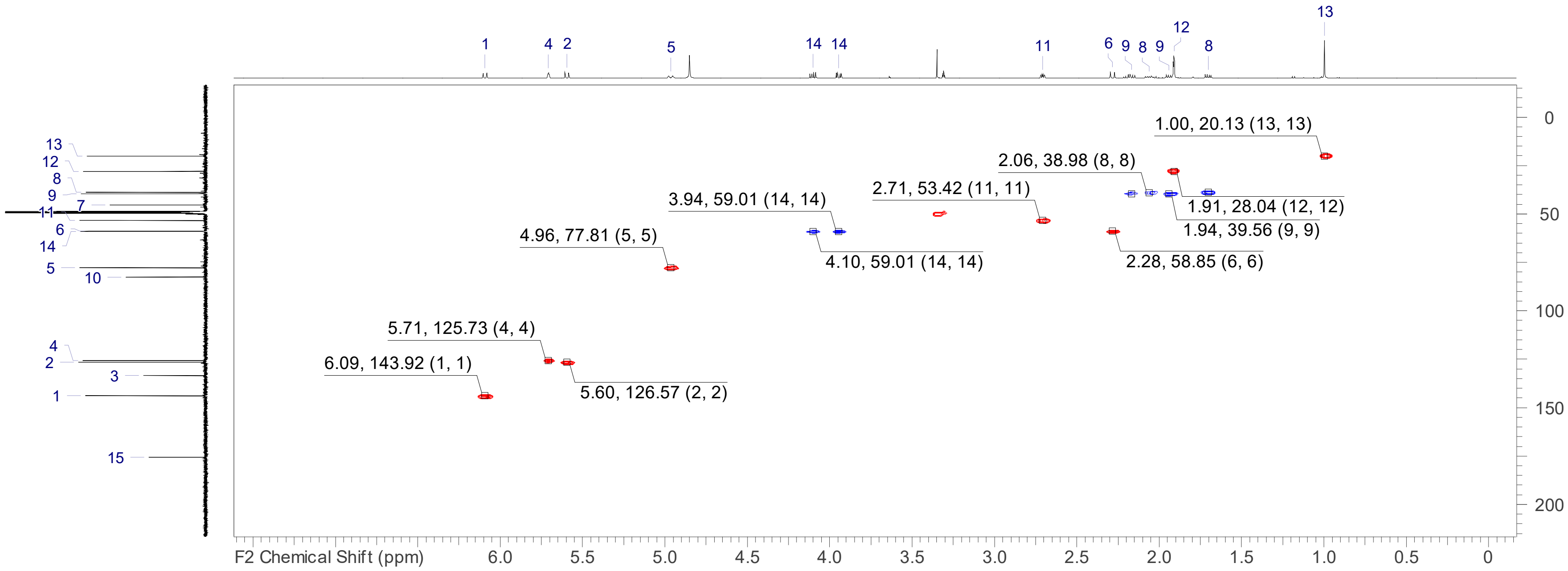

HMBC NMR spectrum (700 MHz, methanol-d_4) of Fulvoferruginin D (**4**).

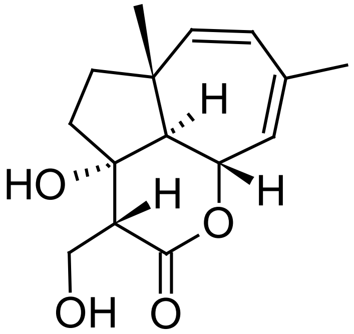

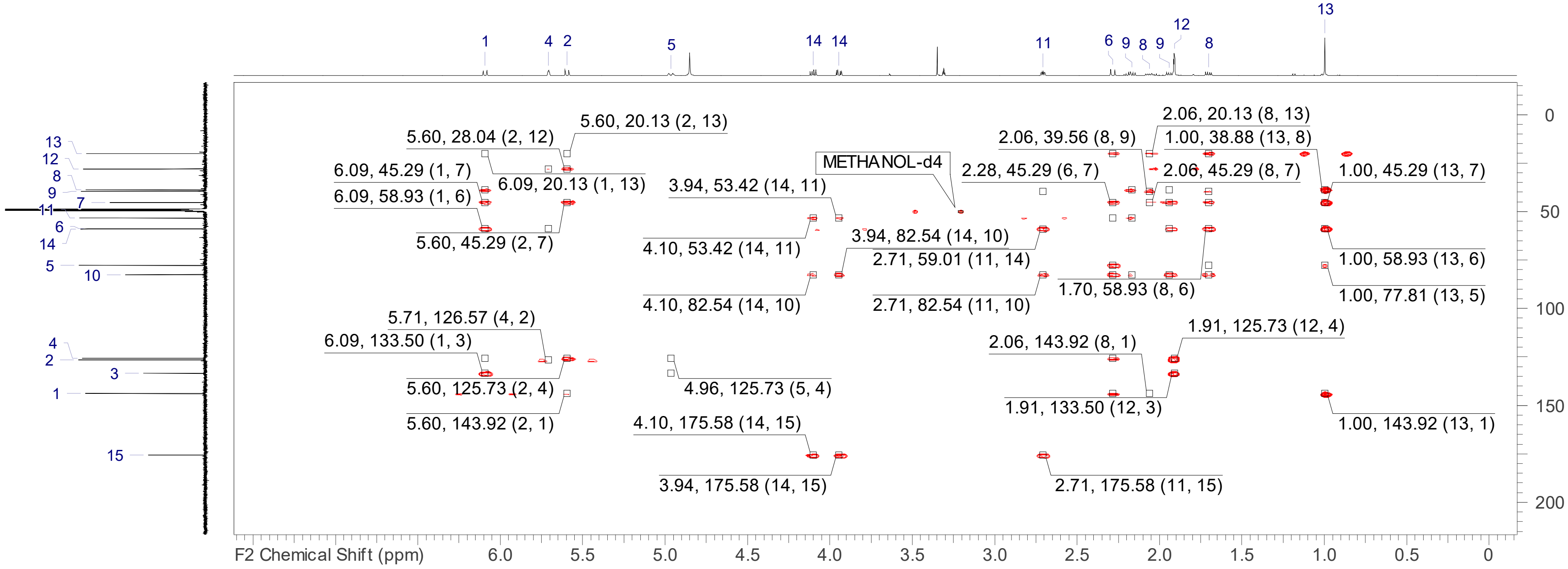

COSY NMR spectrum (700 MHz, methanol-d_4) of Fulvoferruginin D (**4**).

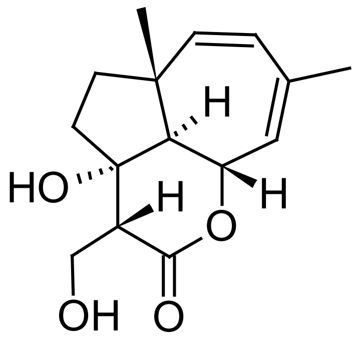

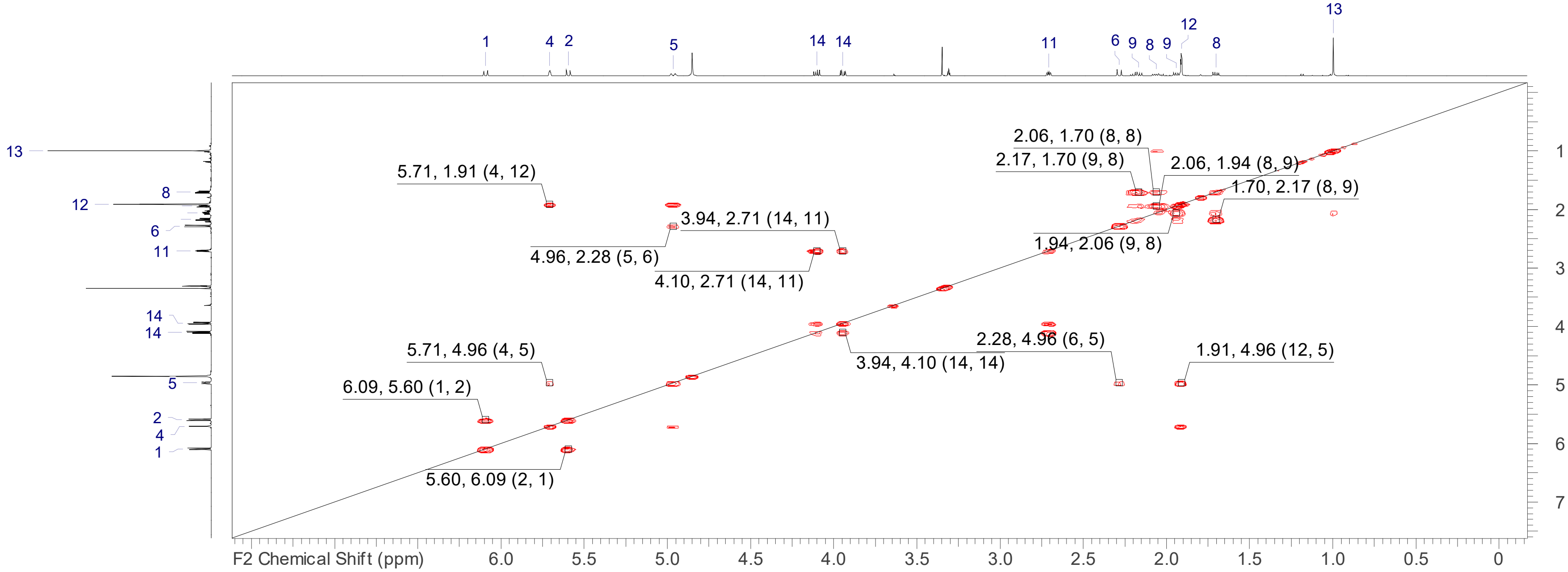

ROESY NMR spectrum (700 MHz, methanol-d_4) of Fulvoferruginin D (**4**).

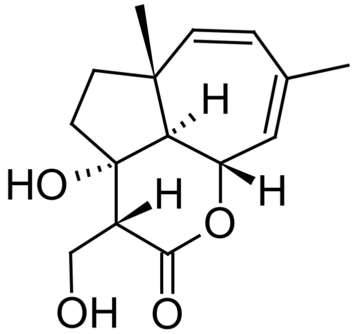

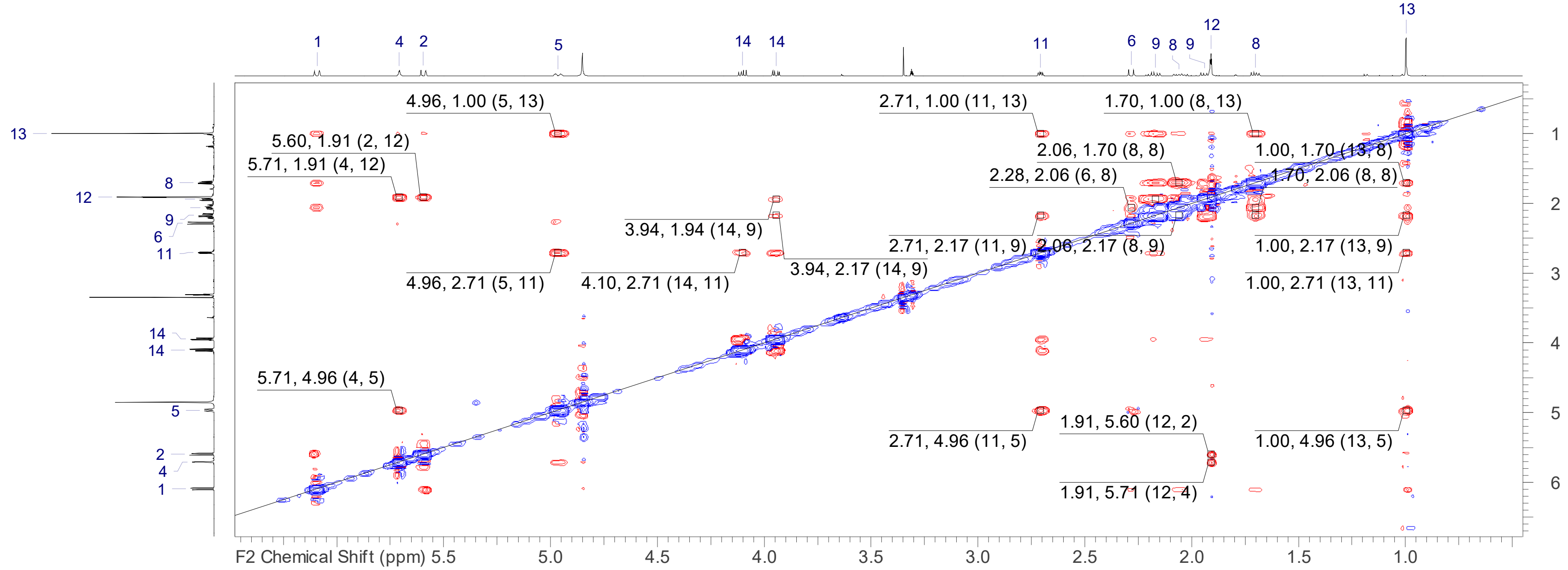

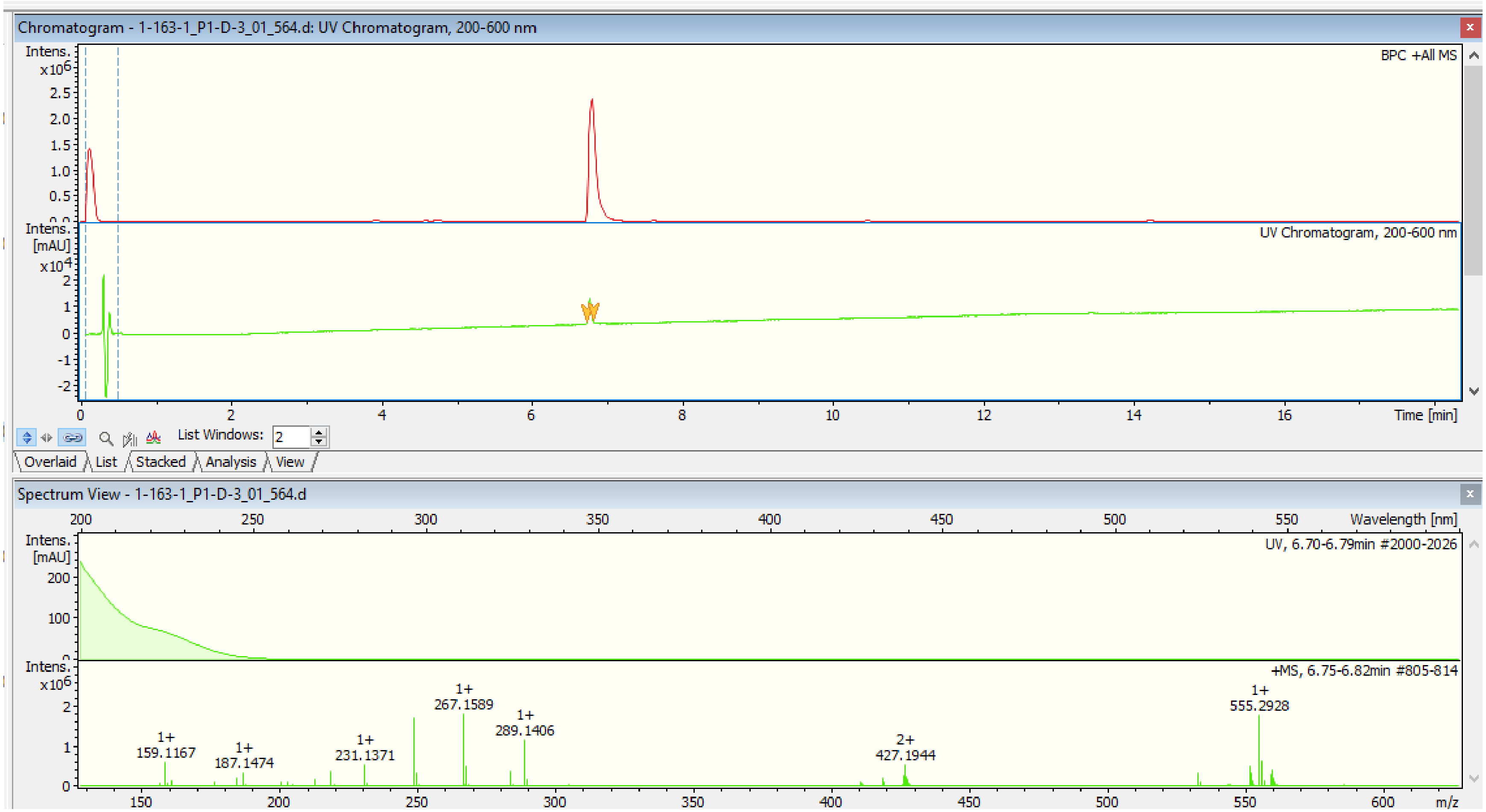

HRESIMS of Fulvoferruginin E (**5**).

^{1}H NMR spectrum (700 MHz, methanol-d_4) of Fulvoferruginin E (**5**).

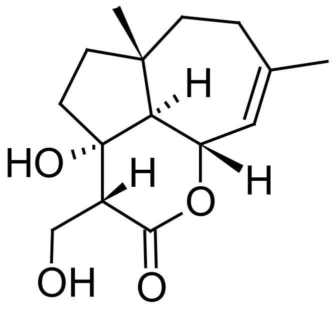

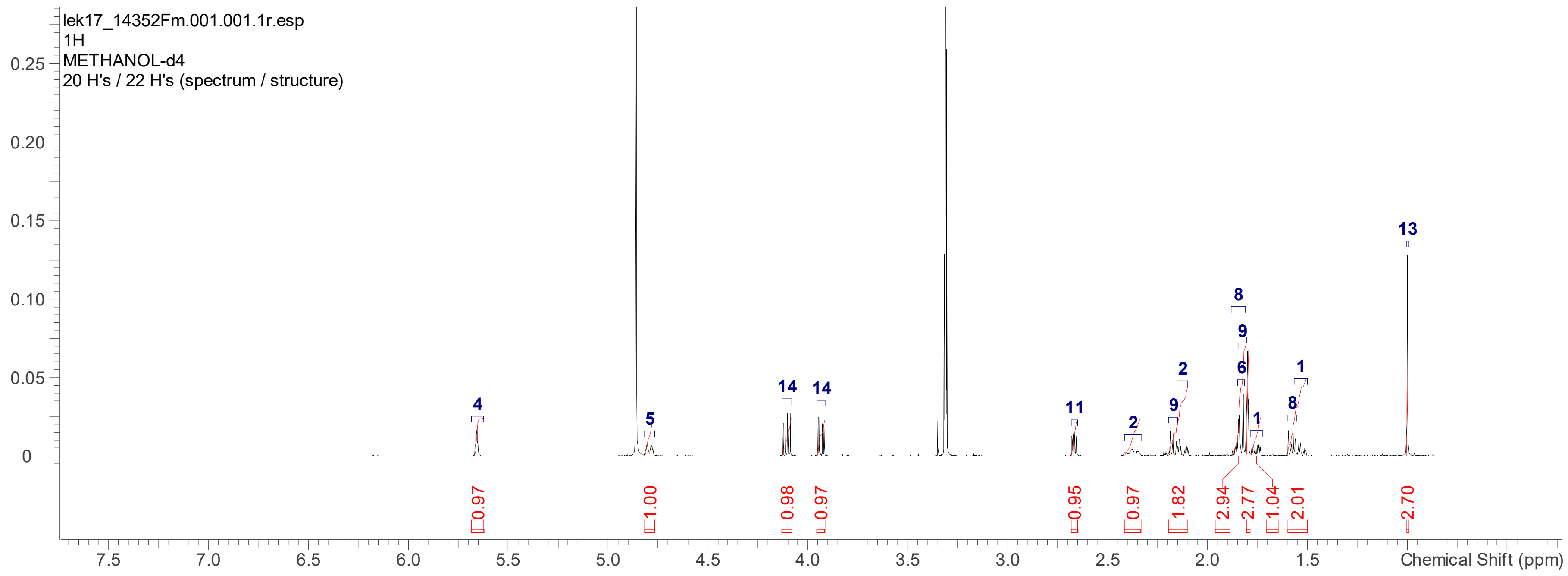

^{13}C NMR spectrum (176 MHz, methanol-d_4) of Fulvoferruginin E (**5**).

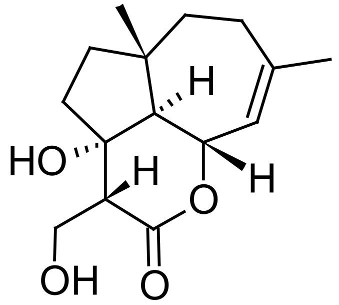

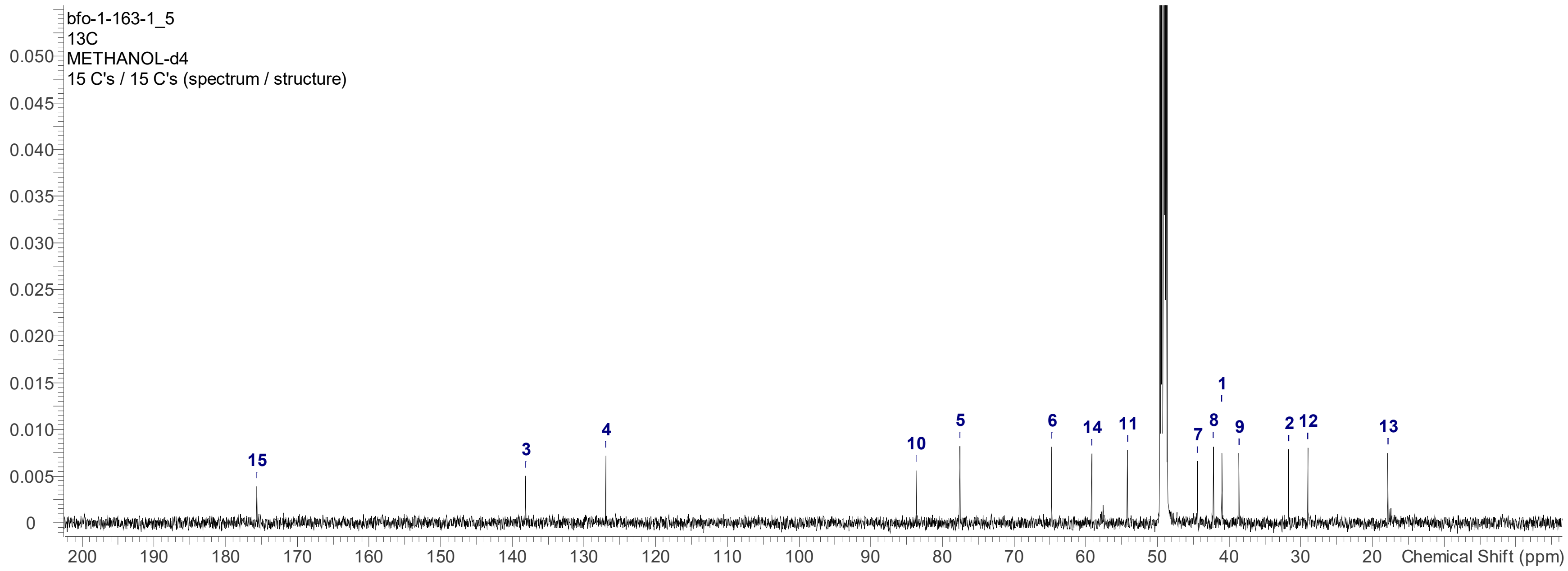

HSQC-dept NMR spectrum (700 MHz, methanol-d_4) of Fulvoferruginin E (**5**).

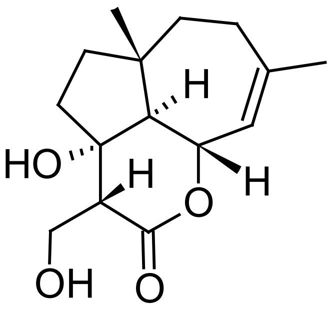

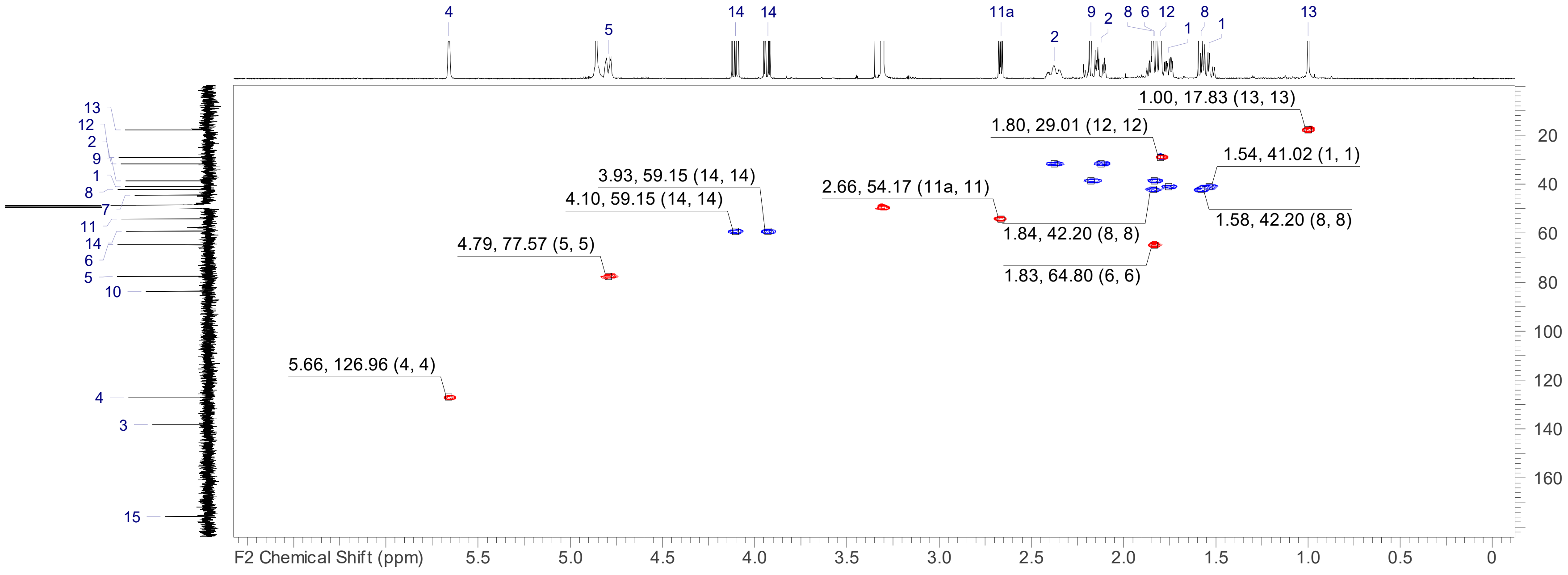

HMBC NMR spectrum (700 MHz, methanol-d_4) of Fulvoferruginin E (**5**).

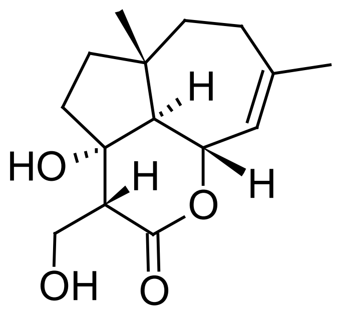

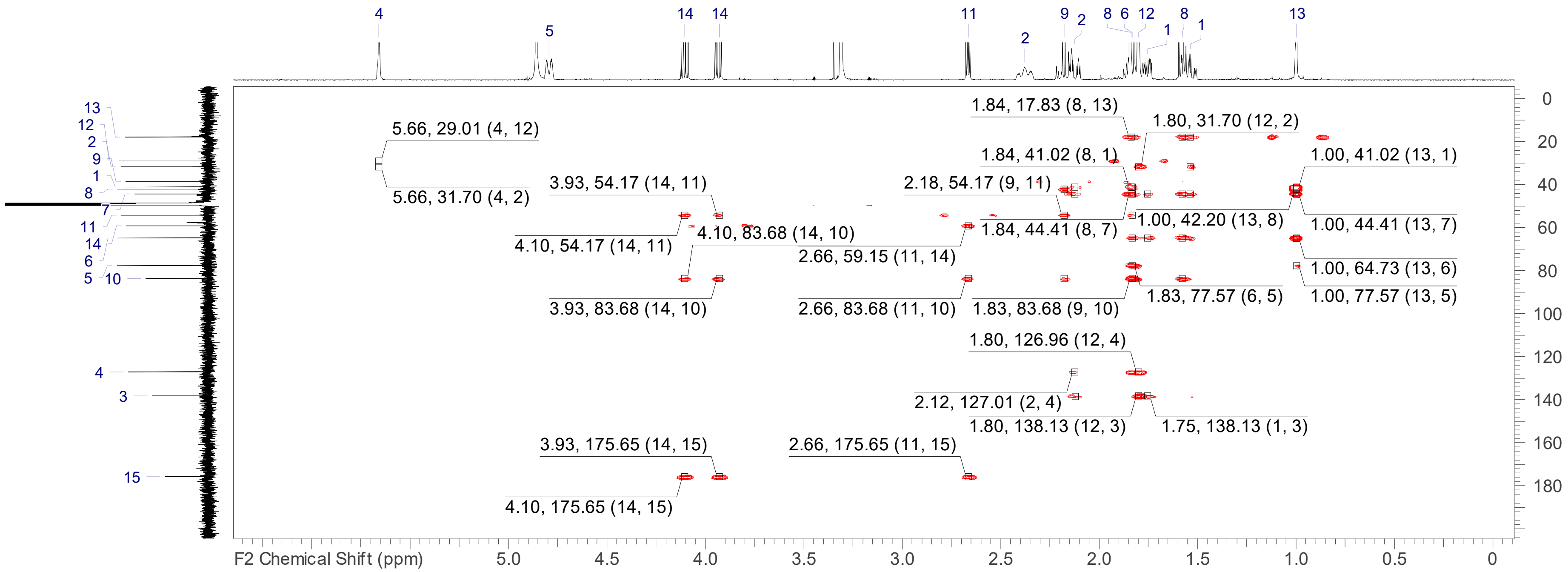

COSY NMR spectrum (700 MHz, methanol-d_4) of Fulvoferruginin E (**5**).

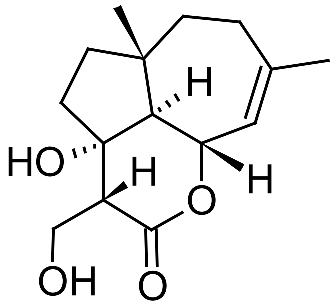

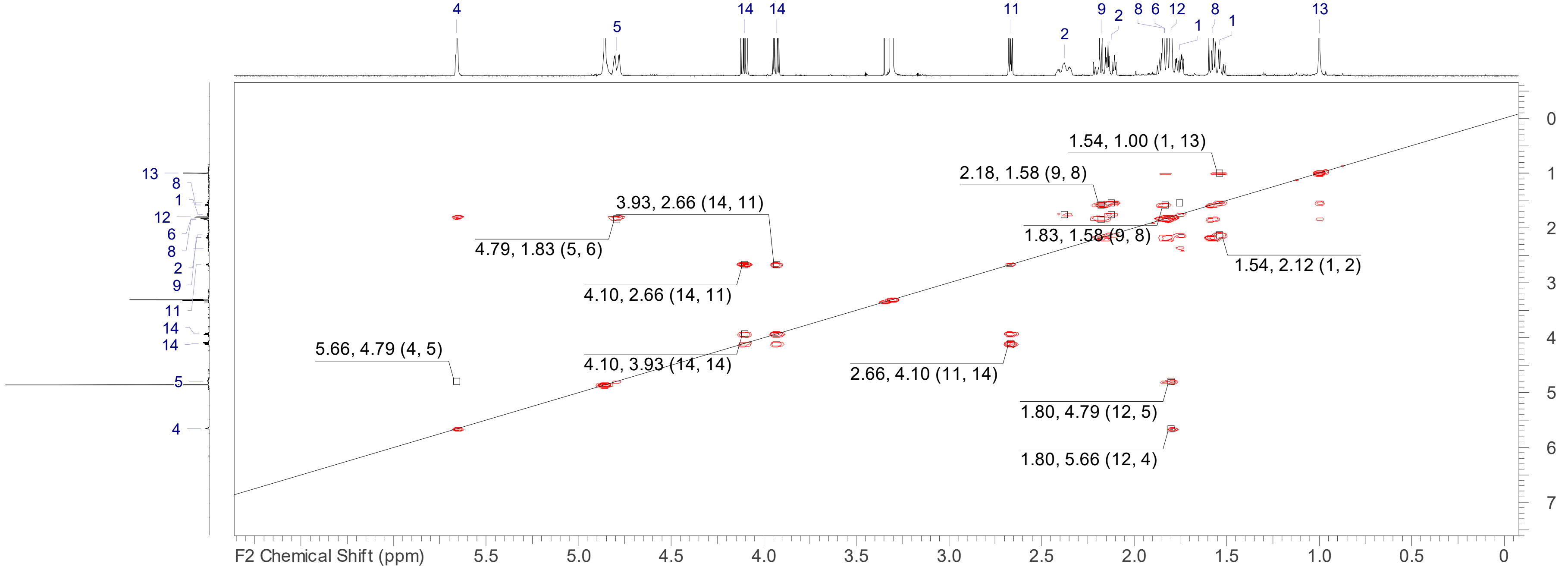

ROESY NMR spectrum (700 MHz, methanol-d_4) of Fulvoferruginin E (**5**).

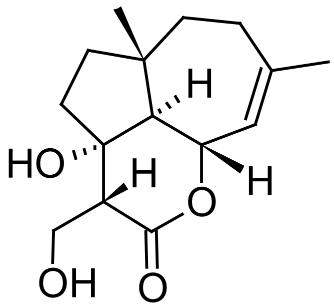

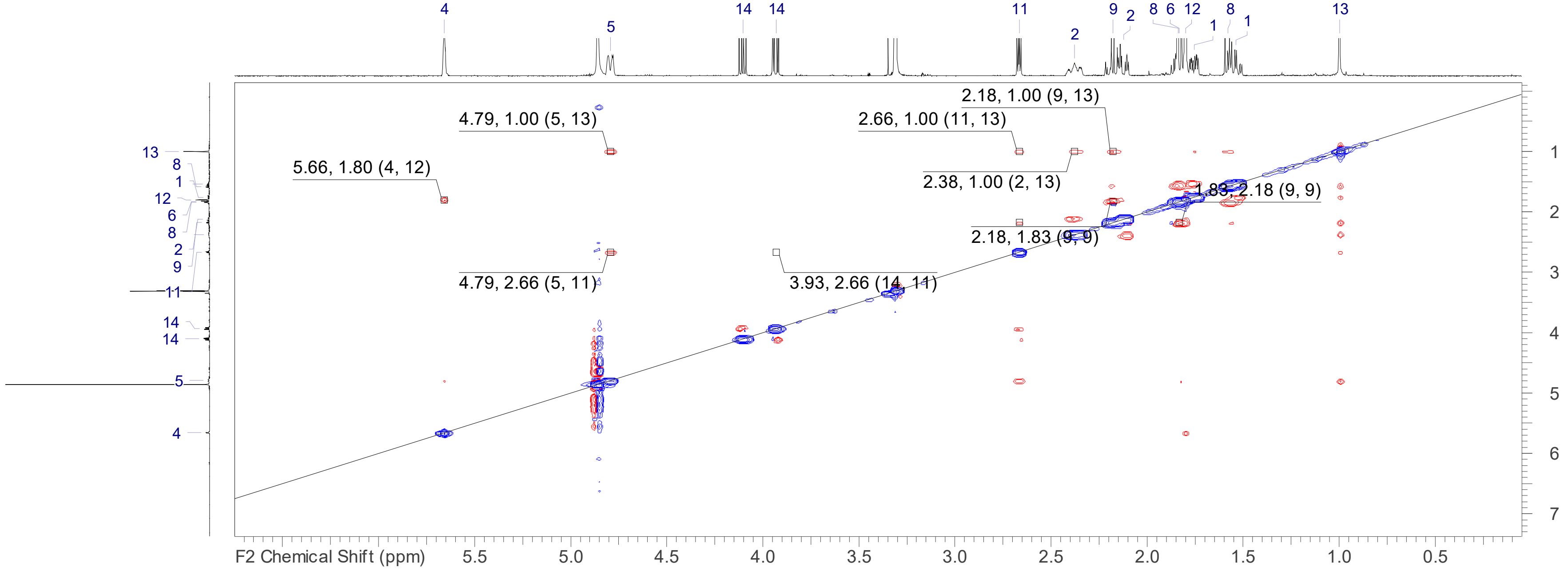

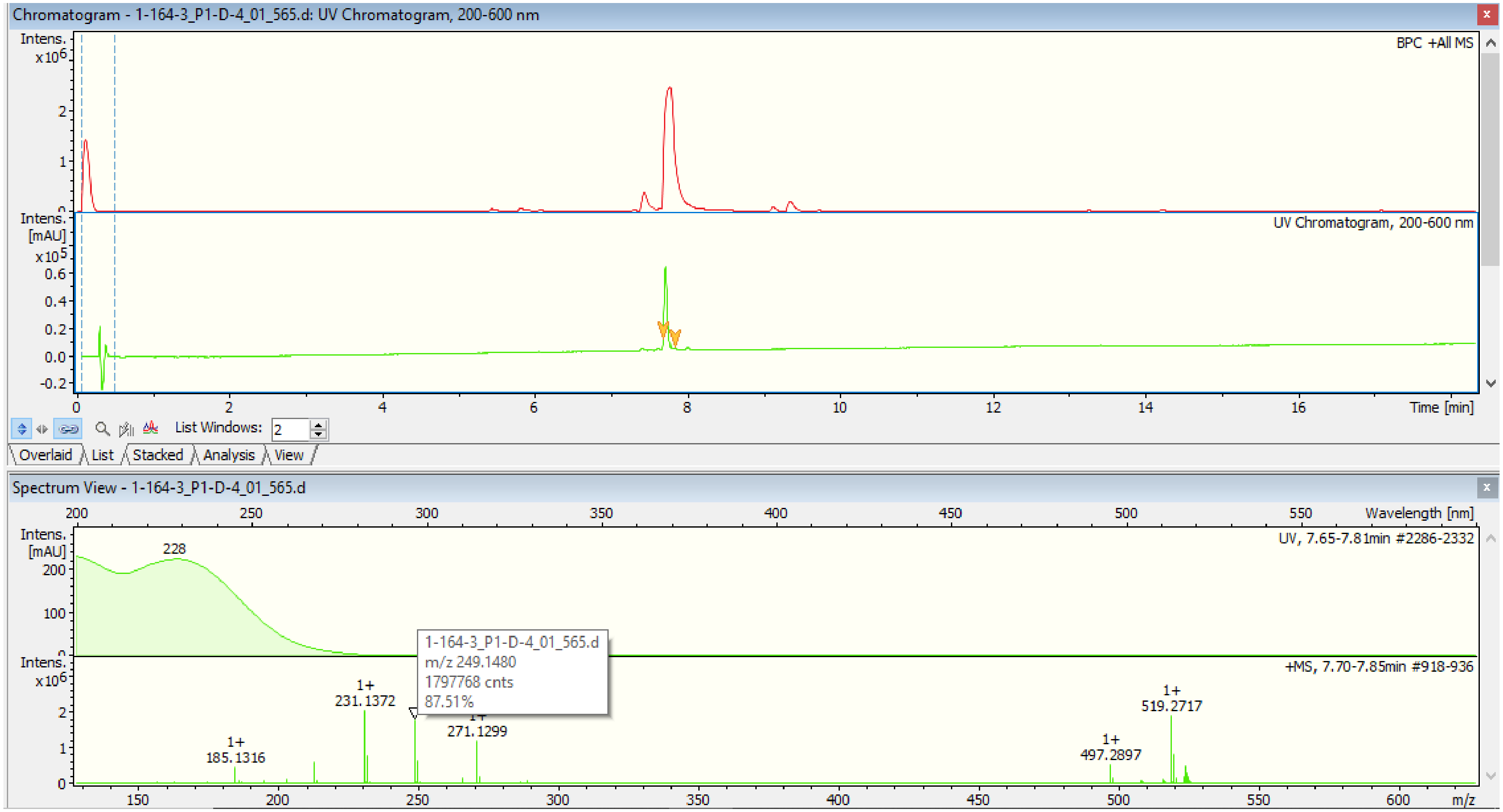

HRESIMS of Fulvoferruginin F (**6**).

^{1}H NMR spectrum (700 MHz, methanol-d_4) of Fulvoferruginin F (**6**).

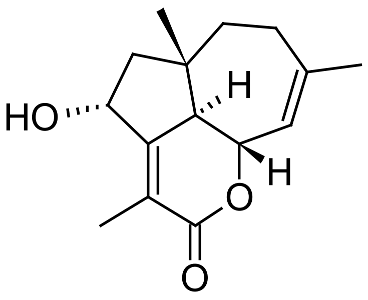

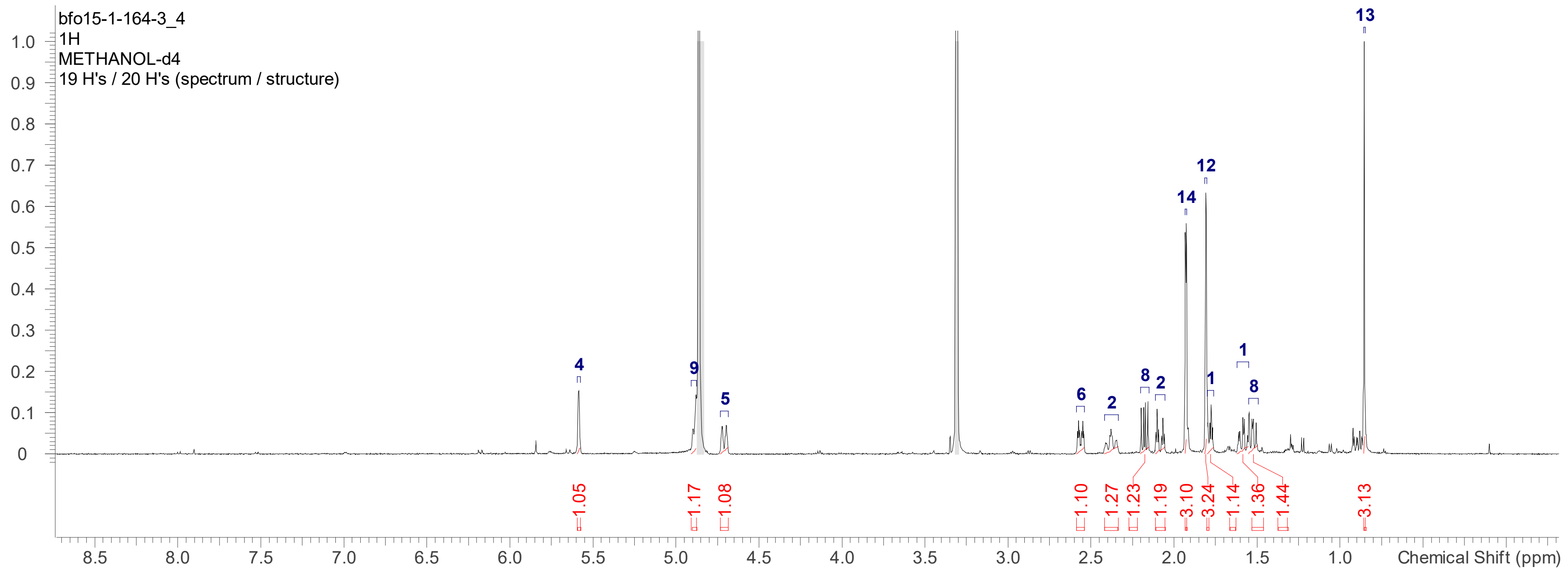

^{13}C NMR spectrum (176 MHz, methanol-d_4) of Fulvoferruginin F (**6**).

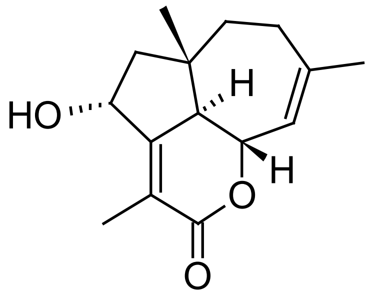

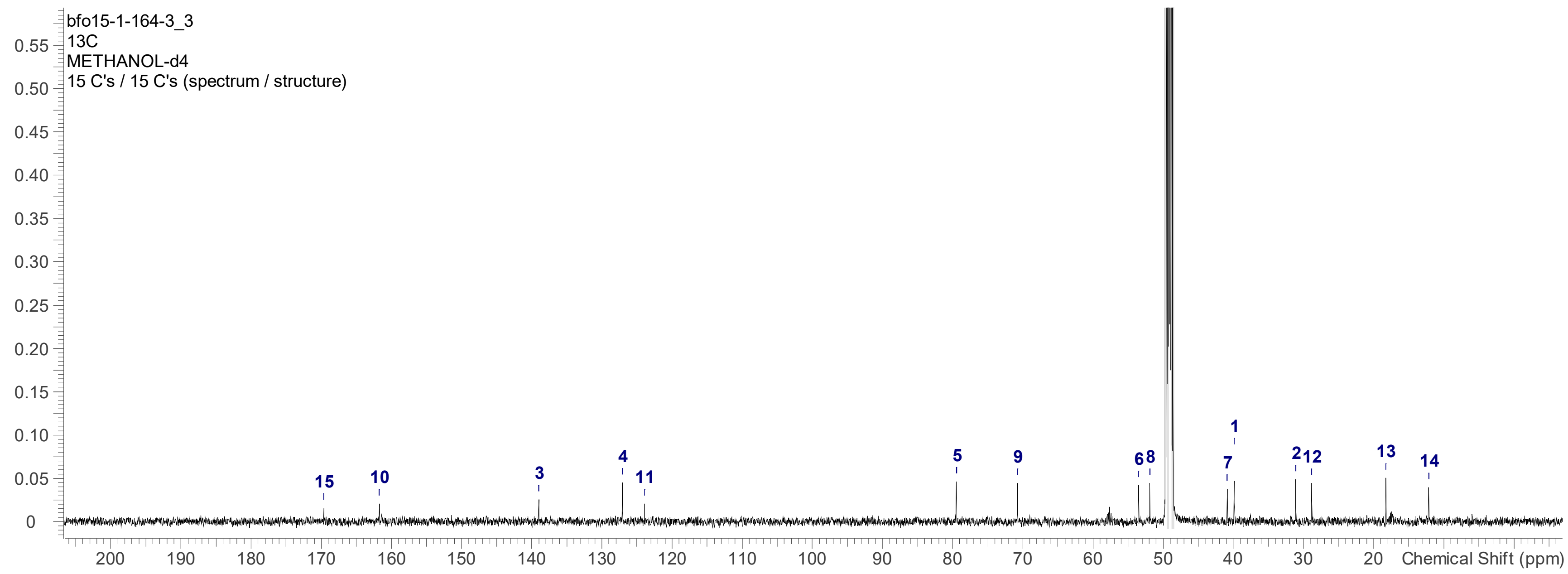

HSQC-dept NMR spectrum (700 MHz, methanol-d_4) of Fulvoferruginin F (**6**).

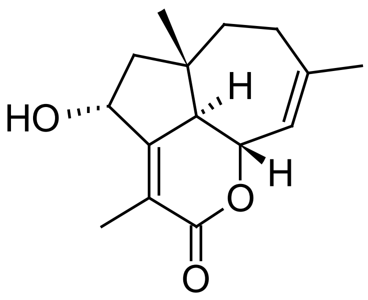

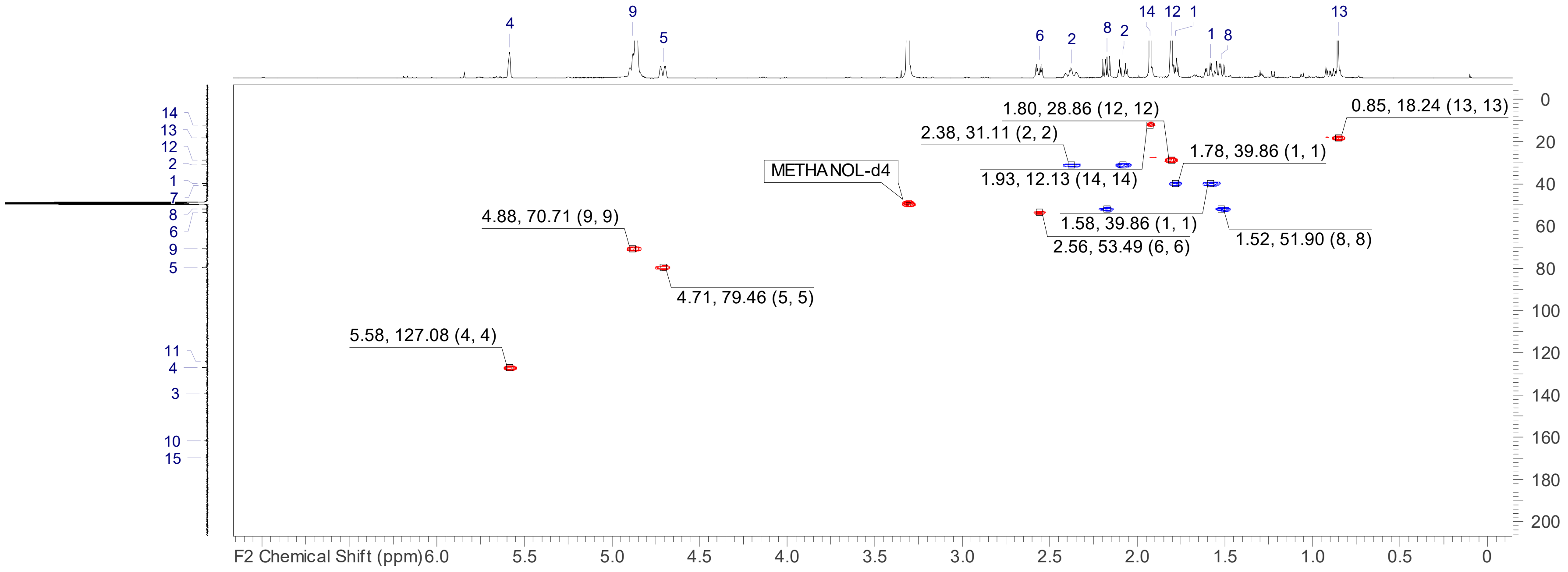

HMBC NMR spectrum (700 MHz, methanol-d_4) of Fulvoferruginin F (**6**).

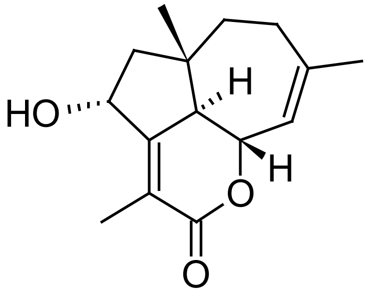

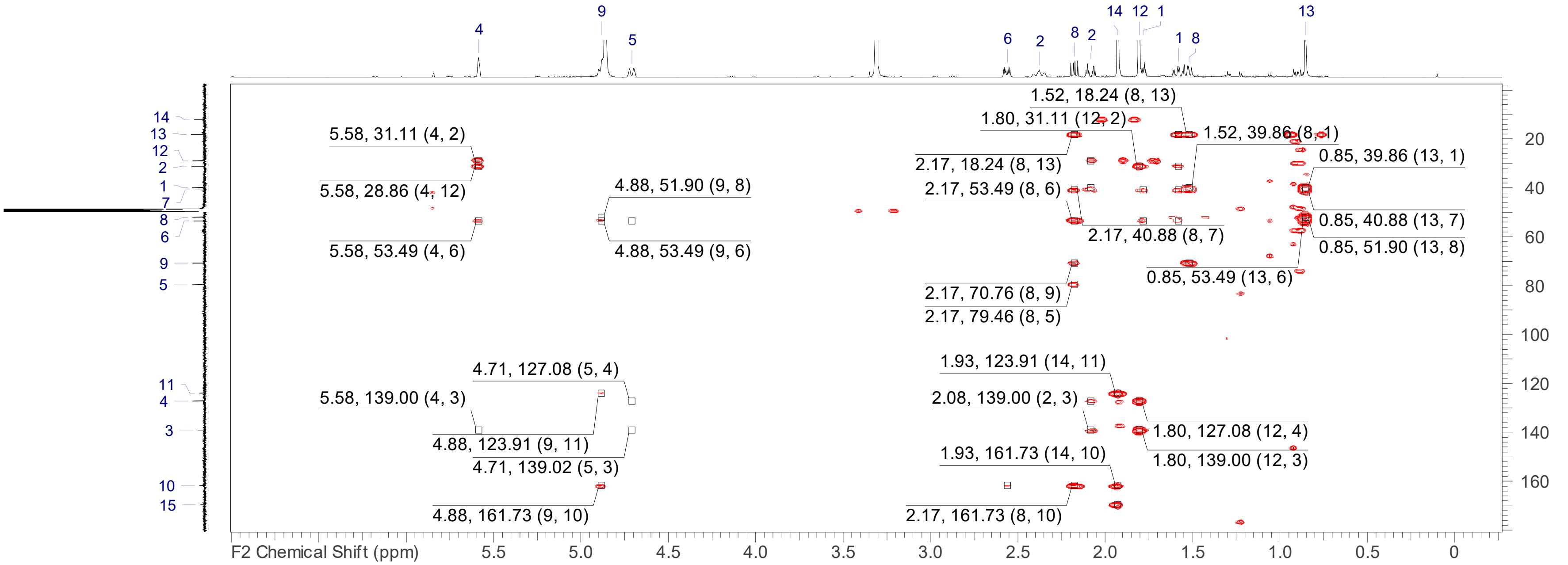

COSY NMR spectrum (700 MHz, methanol-d_4) of Fulvoferruginin F (**6**).

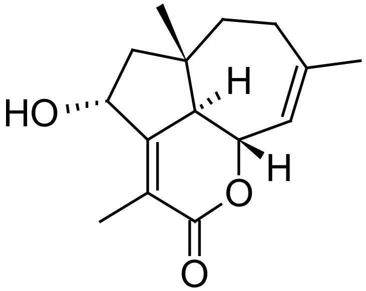

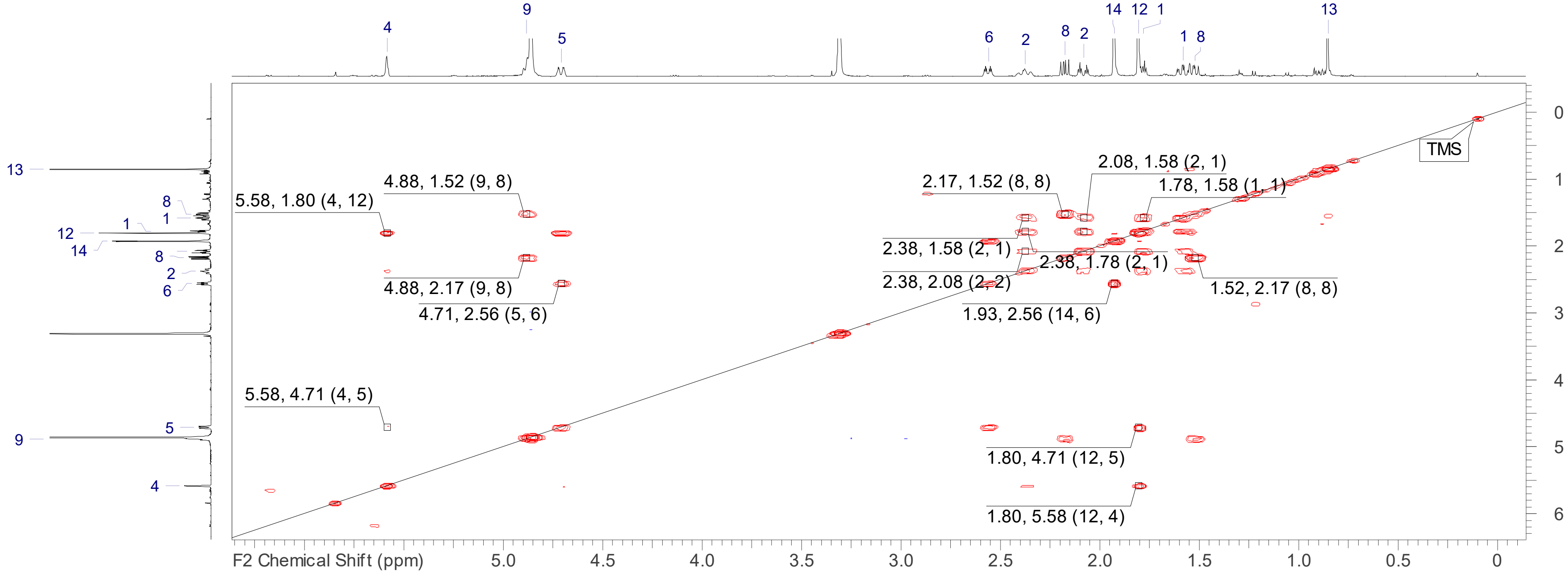

ROESY NMR spectrum (700 MHz, methanol-d_4) of Fulvoferruginin F (**6**).

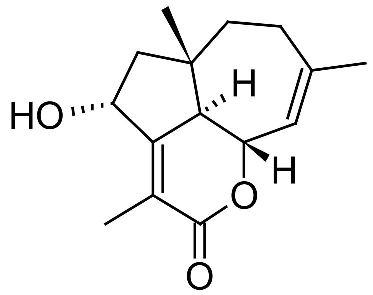

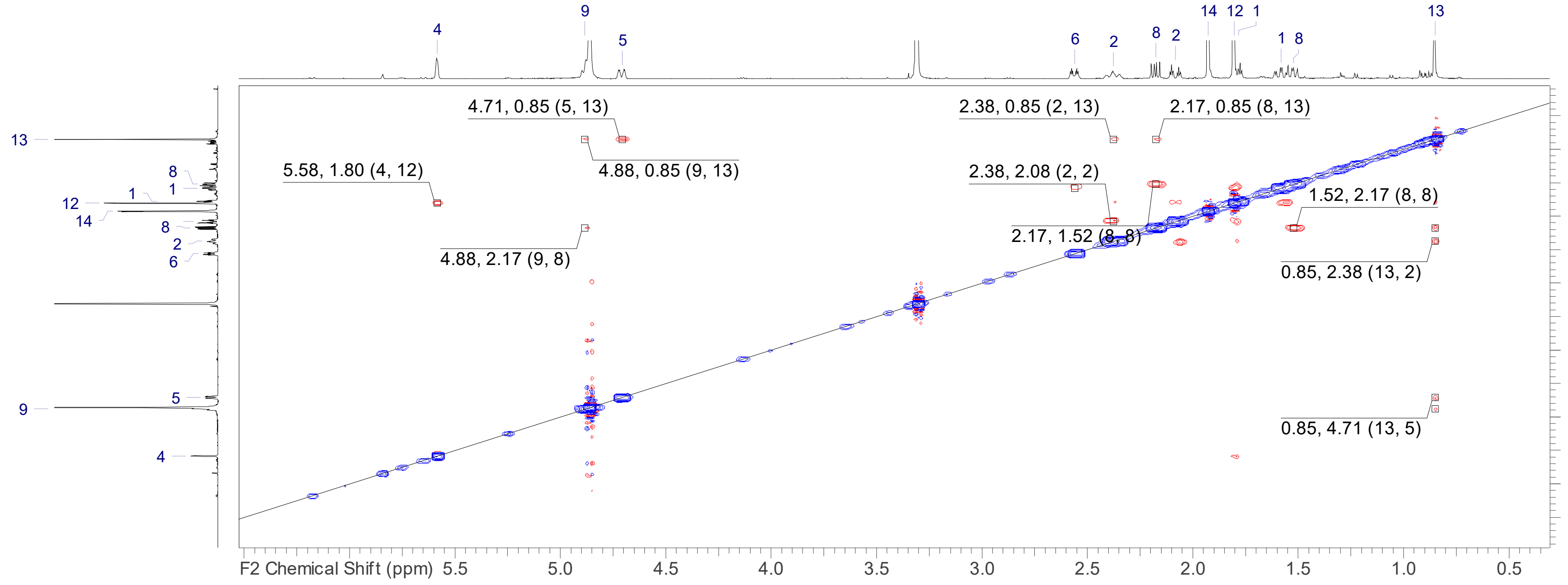

Supplement: File 1 — HRESIMS profiles and copies of NMR spectra for compounds 1–6 in CD3OD, and for metabolite 3 also in CDCl3; minimum inhibitory concentrations (MIC) of 1–6 for bacteria, yeasts and fungi as well as half inhibitory concentrations (IC50) for different cell lines. [file Beilstein_J_Org_Chem-17-1385-s001.pdf]
